# 7β-(3-Ethyl-*cis*-crotonoyloxy)-1α-(2-methylbutyryloxy)-3,14-dehydro-*Z* Notonipetranone Attenuates Neuropathic Pain by Suppressing Oxidative Stress, Inflammatory and Pro-Apoptotic Protein Expressions

**DOI:** 10.3390/molecules26010181

**Published:** 2021-01-01

**Authors:** Amna Khan, Adnan Khan, Sidra Khalid, Bushra Shal, Eunwoo Kang, Hwaryeong Lee, Geoffroy Laumet, Eun Kyoung Seo, Salman Khan

**Affiliations:** 1 Department of Pharmacy, Faculty of Biological Sciences, Quaid-i-Azam University, Islamabad 45320, Pakistan; amna.khn26@gmail.com (A.K.); adkhan165sbbu@gmail.com (A.K.); sidra.merlin@gmail.com (S.K.); bushra.shal@gmail.com (B.S.); 2 College of Pharmacy, Graduate School of Pharmaceutical Sciences, Ewha Womans University, Seoul 03760, Korea; smileunu@gmail.com (E.K.); jongsky119@naver.com (H.L.); 3 Department of Physiology, Michigan State University, East Lansing, MI 48824, USA; laumetge@msu.edu

**Keywords:** neuropathic markers, anti-neuroinflammation, ECN, myelin sheath, DNA disruption

## Abstract

7β-(3-Ethyl-*cis*-crotonoyloxy)-1α-(2-methylbutyryloxy)-3,14-dehydro-*Z*-notonipetranone (ECN), a sesquiterpenoid obtained from a natural source has proved to be effective in minimizing various side effects associated with opioids and nonsteroidal anti-inflammatory drugs. The current study focused on investigating the effects of ECN on neuropathic pain induced by partial sciatic nerve ligation (PSNL) by mainly focusing on oxidative stress, inflammatory and apoptotic proteins expression in mice. ECN (1 and 10 mg/kg, i.p.), was administered once daily for 11 days, starting from the third day after surgery. ECN post-treatment was found to reduce hyperalgesia and allodynia in a dose-dependent manner. ECN remarkably reversed the histopathological abnormalities associated with oxidative stress, apoptosis and inflammation. Furthermore, ECN prevented the suppression of antioxidants (glutathione, glutathione-S-transferase, catalase, superoxide dismutase, NF-E2-related factor-2 (Nrf2), hemeoxygenase-1 and NAD(P)H: quinone oxidoreductase) by PSNL. Moreover, pro-inflammatory cytokines (tumor necrotic factor-alpha, interleukin 1 beta, interleukin 6, cyclooxygenase-2 and inducible nitric oxide synthase) expression was reduced by ECN administration. Treatment with ECN was successful in reducing the caspase-3 level consistent with the observed modulation of pro-apoptotic proteins. Additionally, ECN showed a protective effect on the lipid content of myelin sheath as evident from FTIR spectroscopy which showed the shift of lipid component bands to higher values. Thus, the anti-neuropathic potential of ECN might be due to the inhibition of oxidative stress, inflammatory mediators and pro-apoptotic proteins.

## 1. Introduction

Neuropathic pain can result from the direct injury to various peripheral nerves via peripheral and central sensitization which leads to impaired pain processing [1,2,3]. Neuropathic pain can arise from traumatic spinal cord injury, multiple sclerosis, stroke, persistent diabetes, lumbar disc syndrome, herpes infection, cancer, and AIDS [4,5]. Neuropathic pain is characterized by burning and stabbing pain due to hyperactivity of nerve fibers leading to hyperalgesia (increased pain sensitivity) and allodynia (pain resulting from non-painful stimuli) [6,7,8].

Management of neuropathic pain remains a major clinical challenge due to an inadequate understanding of the mechanism involved in the pathogenesis of neuropathic pain [9,10]. Presently, most of the available medications used against neuropathic pain such as anti-convulsant, anti-depressants, opioids, N-methyl D-aspartate (NMDA) receptor agonists, sodium channel blockers, and cannabinoids receptor agonist are often accompanied by dose-limiting side effects [11,12]. Up to now, none of the medications are effective in neuropathic pain and have been failed in terms of effectiveness and safety [13,14]. In this context, natural products that present fewer side effects emerge as interesting therapeutic resources for the development of new drugs for the management of chronic pain [15,16,17]. Therefore, the development and utilization of more effective analgesic agents of natural origin that suppressed neuropathic pain remain desired.

Oxidative stress and inflammation play a key role in the initiation and maintenance of neuropathic pain [18,19,20]. Oxidative stress leads to the activation of reactive oxygen species which as a result amplifies the pathogenesis of neuropathic pain [21,22,23]. It has been reported that the underlying cause of oxidative stress-mediated neuropathic pain is the under-expression of Nrf2 [24,25,26], a transcription factor involved in the regulation of genes that encodes antioxidant proteins and phase 2 detoxifying enzymes [27,28,29]. Nrf2 binding with the antioxidant responsive element (ARE) leads to the transcriptional activation of downstream genes such as NAD(P)H: quinone oxidoreductase (NQO1), glutathione-S-transferase (GST), and hemeoxygenase-1 (HO-1) [27,30,31,32]. Moreover, several studies have shown that overexpression of Nrf2 produces a protective effect against oxidative stress [18,33,34,35]. Recently, the activation of inflammatory markers and apoptotic pathways in the pathogenesis of neuropathic pain has been demonstrated [36,37,38]. Expression of inflammatory mediators such as tumor necrotic factor-alpha (TNF-α), interleukin 1 beta (1L-1β), interleukin 6 (1L-6), cyclooxygenase-2 (COX-2), inducible nitric oxide synthase (iNOS) while pro-apoptotic protein such as (caspase 3) contributes to the induction and maintenance of neuropathic pain [39,40,41,42].

*Tussilago farfara* is a perennial medicinal plant that belongs to the Asteraceae family. Previous phytochemical investigations on *T. farfara* revealed the presence of sesquiterpenoids, pyrrolizidine alkaloids, terpenoids, chromones and flavonoids [43,44,45]. Jang et al. described the bioassay-guided isolation of a sesquiterpene and chromone along with 18 known compounds from the flowering buds of *T. farfara* [46]. Preliminary bioactivity screening showed that the methanolic extract of *T. farfara* exhibits an inhibitory effect on LPS-induced nitric oxide production [46,47]. It was also found that ethyl acetate fraction of *T. farfara* potently inhibited neuronal damage induced by arachidonic acid [48]. Further reported that bioassay-guided ethanolic extract of *T. farfara* inhibited microsomal diacylglycerol acyltransferase 1 derived from rat liver and human hepatocellular carcinoma HepG2 cells [49]. It also significantly inhibited triglyceride synthesis by suppressing the incorporation of label [^14^C] acetate or [^14^C] glycerol into triglycerides in HepG2 cells [49]. A pure compound of *T. farfara*, ECN has been reported to activate Nrf2/HO-1 signaling pathway in both PC12 cells and mice [50]. A previous study revealed that ECN exhibits anti-inflammatory actions in activated microglia and cytoprotective effects against LPS induced neuronal cell death [51]. Further ECN has been reported to inhibit JAK-STAT3 signaling and expression of STAT3 targeted genes [52]. Because oxidative stress and inflammation are key mechanisms for neuropathic pain, the aim of present study was to assess the anti-neuropathic activities of ECN through the reduction of expression of oxidative stress proteins, inflammatory markers (COX-2, iNOS, TNF-α, 1L-1β, 1L-6), and pro-apoptotic protein (caspase-3) by PSNL in mice.

## 2. Results

### 2.1. Effect of ECN on Distress Symptoms and Survival Rate

**Figure 1:**
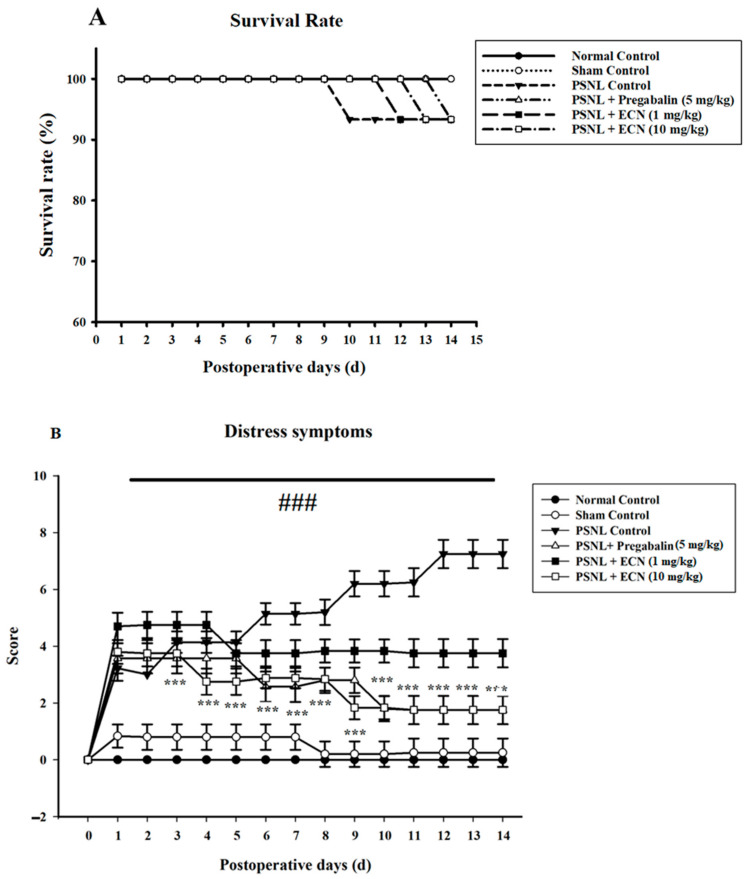
Effect of ECN treatment on survival rate and distress symptoms. (**A**) Survival rate during the experiment. Data are presented as a percentage of number of mice survived each day. (**B**) Effect of ECN (1 and 10 mg/kg i.p.) treatment on distress symptoms. The total score of distress symptoms (dull/ruffled coat, change in temperament, reluctance to move) were recorded daily. Each criteria was scored from (0–3). The *p* > 0.001 value has been calculated from actual raw counts by applying two-way ANOVA followed by Dunnett’s t-tests. The average and standard deviation of actual counts were expressed in the percentages using the standard formula. Different letters meant statistically significant differences: (***) *p* < 0.001 indicate significant differences from the PSNL control group. (###) indicate significant differences from the normal control group. *** *p* < 0.001 (two-way ANOVA followed by Dunnett’s test).

The survival results showed that more than 90 percent of mice were survived during partial sciatic nerve ligation (PSNL) compared to normal control (Figure 1A). The PSNL treated group showed the highest distress score as compared to the normal group (*p* < 0.05). The ECN treatment groups displayed reduced distress scores as compared to the PSNL treated group (*p* < 0.001) (Figure 1B).

### 2.2. Effect of ECN on Neuropathic Mechanical Hyperalgesia, Thermal Hyperalgesia, Mechanical Allodynia and Cold Allodynia

**Figure 2:**
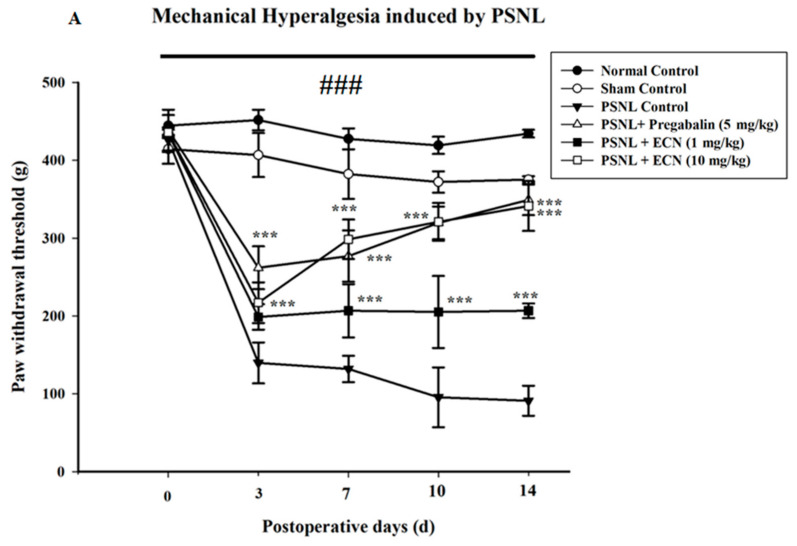

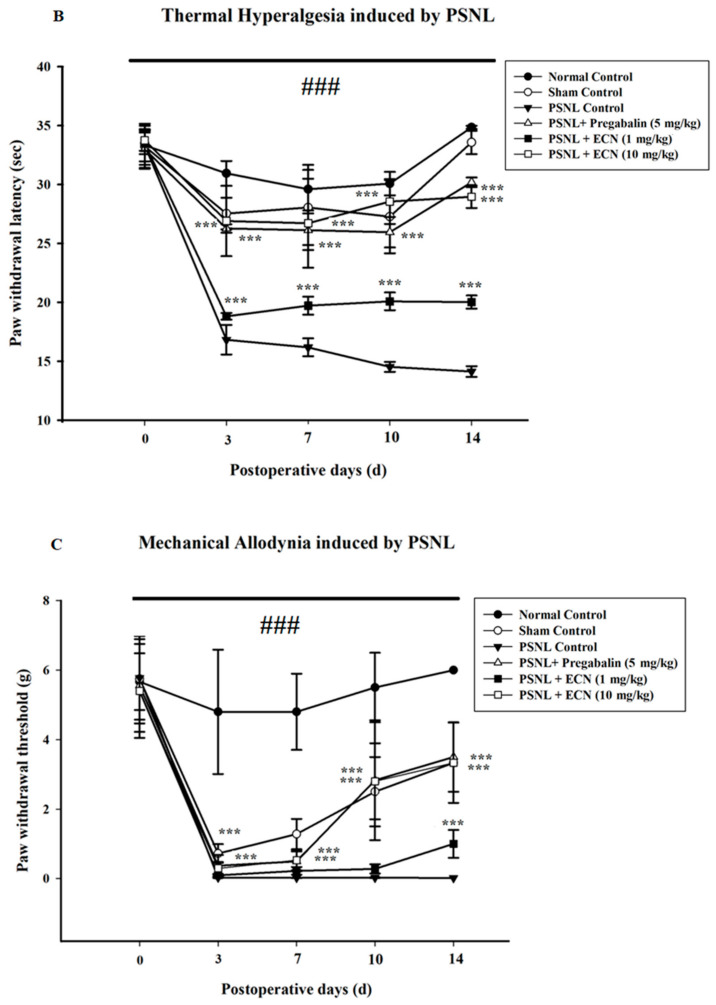

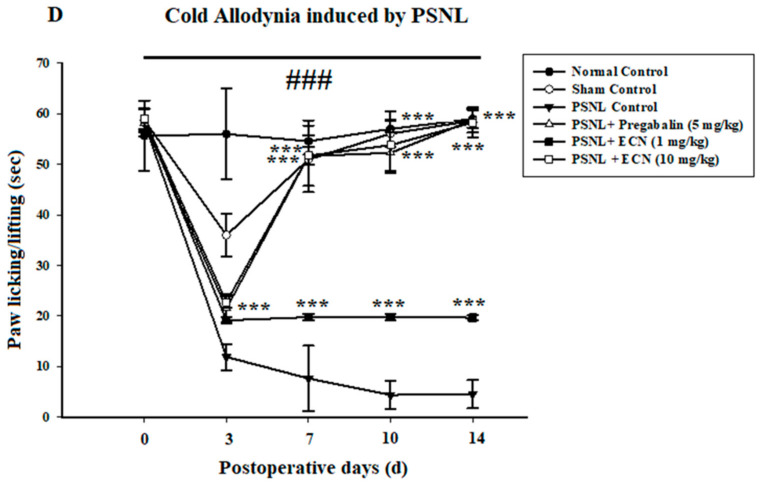
Effect of ECN treatment on mechanical hyperalgesia, thermal hyperalgesia, mechanical allodynia and cold allodynia. (**A**) Inhibition of PSNL-induced mechanical hyperalgesia at 0, 3, 7, 10 and 14-day intervals by ECN (1 and 10 mg/kg i.p.). (**B**) Inhibition of PSNL-induced thermal hyperalgesia at 0, 3, 7, 10 and 14-day intervals by ECN (1 and 10 mg/kg i.p). (**C**) Inhibition of PSNL-induced mechanical allodynia at 0, 3, 7, 10 and 14-day intervals by ECN (1 and 10 mg/kg i.p). (**D**) Inhibition of PSNL-induced cold allodynia at 0, 3, 7, 10 and 14-day intervals by ECN (1 and 10 mg/kg i.p). The data were reported as the means ± S.D. Different letters meant statistically significant differences: (***) *p* < 0.001 indicate significant differences from the PSNL control group. (###) indicate significant differences from the normal control group. *** *p* < 0.001 (two-way ANOVA followed by Dunnett’s test).

In PSNL-induced hyperalgesia, post-treatment with ECN (10 mg/kg) significantly (F = 34.4, *p* < 0.001) inhibited mechanical hyperalgesia on day 3 in a dose-dependent manner as compared to PSNL control (Figure 2A). Further evaluation of anti-inflammatory activity of drug was carried out by evaluating thermal hyperalgesia. Three days after injury, thermal hyperalgesia was significantly reduced in treatment groups as compared to the PSNL-treated group. Post-treatment with ECN (10 mg/kg) significantly (F = 44.7, *p* < 0.001) reduced the hyperalgesic responses such as paw licking and jumping (Figure 2B). Seven days after injury, thermal and mechanical hyperalgesia were still present in neuropathic mice, whereas ECN and positive control reversed the nociceptive hypersensitivity. In PSNL-induced mechanical allodynia, post-treatment with ECN (10 mg/kg) significantly (F = 27.6, *p* < 0.001) reduced mechanical allodynia as compared to the PSNL-treated group (Figure 2C). In PSNL-induced cold allodynia, post-treatment with ECN (10 mg/kg) inhibited (F = 53.5, *p* < 0.001) cold allodynia as compared to PSNL-treated group (Figure 2D). In brief, ECN reduced the nociceptive hypersensitivity because of the neuropathic lesion in a time and dose-dependent manner.

### 2.3. Effect of ECN on Neuropathic Muscle and Motor Coordination

**Figure 3:**
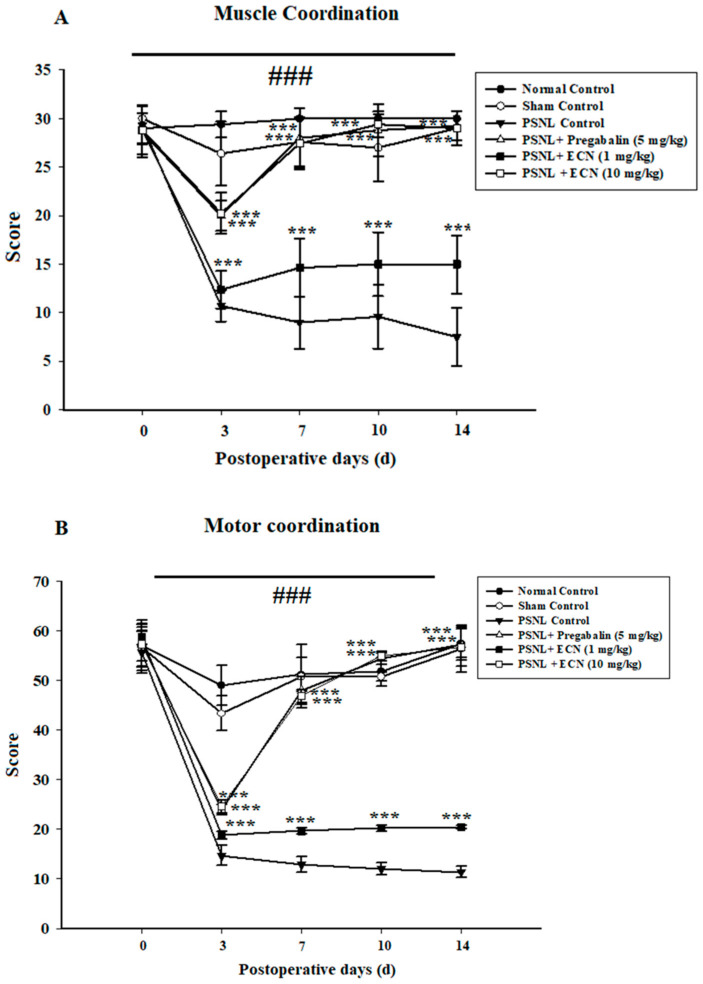
Effect of ECN (1 and 10 mg/kg i.p.) on PSNL-induced muscle activity by (**A**) Muscle coordination (**B**) Motor coordination. The effect on muscle coordination and motor coordination was measured at 0, 3, 7, 10 and 14 day after PSNL. The data were reported as the means S.D; (n = 5 mice per group) of scoring average. Different letters meant statistically significant differences: (***) *p* < 0.001 indicate significant differences from the PSNL control group. (###) indicate significant differences from the normal control group. *** *p* < 0.001 (two-way ANOVA followed by Dunnett’s test).

Weight lifting and inverted screen tests showed that ECN (10 mg/kg) significantly inhibited PSNL-induced deficit in muscle (F = 84.5, *p* < 0.001) as well as motor coordination (F = 77.4, *p* < 0.001) (Figure 3).

### 2.4. Effect of ECN on DNA Disruption

**Figure 4:**
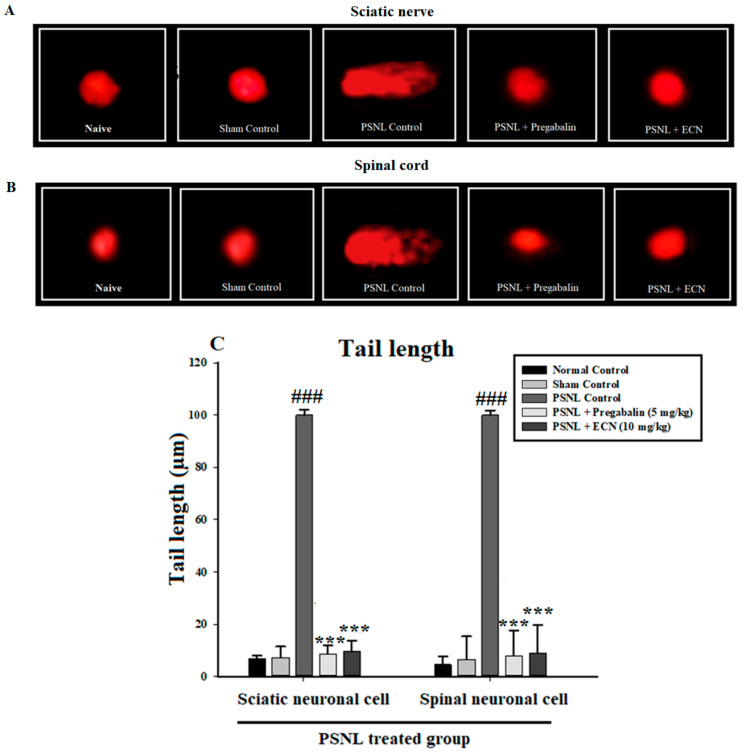

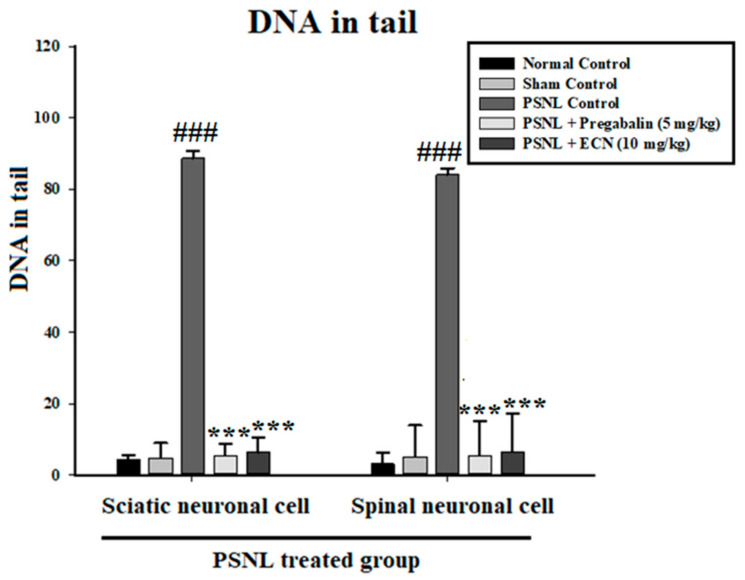
Effect of ECN on DNA damage in the sciatic nerve and spinal cord tissues analysed by comet assay. Fluorescence photomicrographs exhibiting the protective effect of ECN against PSNL induced DNA damage in (**A**) sciatic nerve and (**B**) spinal cord. (**C**) DNA content is estimated by tail length and % DNA in the tail in the PSNL experiment. The data is presented as the mean (n = 5) ± S.D. Different letters meant statistically significant differences: (***) *p* < 0.001 indicate significant differences from the PSNL control group. (###) indicate significant differences from the normal control group. *** *p* < 0.001 (one-way ANOVA followed by Bonferroni post-test).

PSNL control group showed augmentation of DNA damage in the sciatic nerve and spinal cord tissue. Administration of ECN (10 mg/kg) significantly (*p* < 0.001) reduced DNA damage with a decrease in tail length and percentage DNA in the tail as compared to the PSNL challenged group (Figure 4).

### 2.5. Effect of ECN on Histological Examination

**Figure 5:**
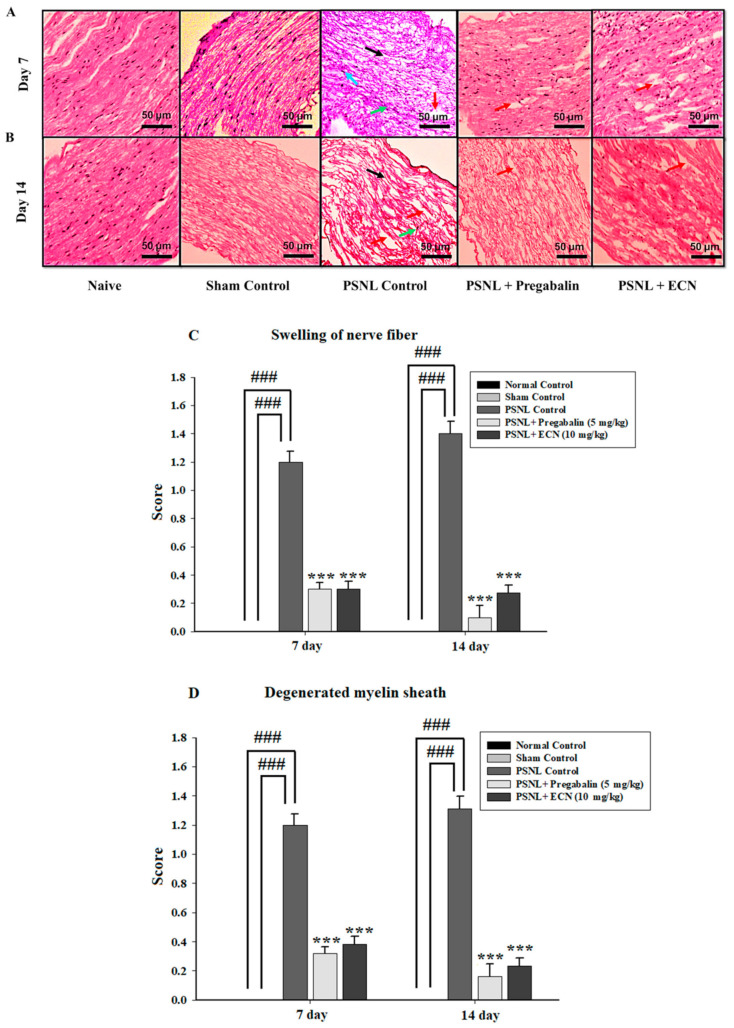

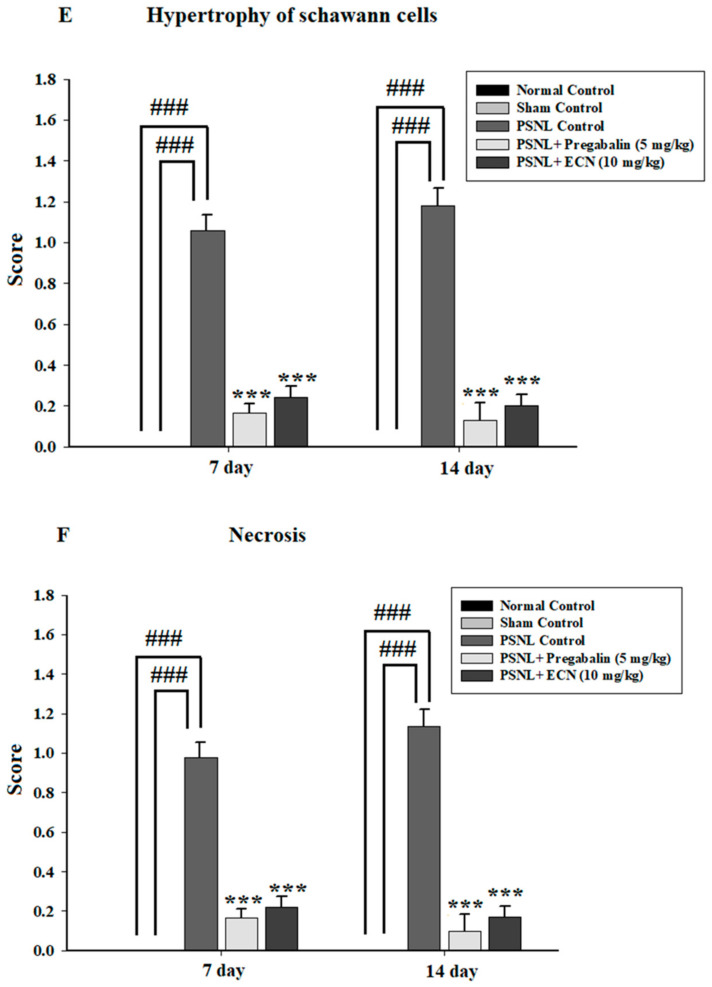
Effect of ECN (10 mg/kg) on the pathological histology of sciatic nerve tissue. The sciatic nerve of mice subjected to PSNL is shown in longitudinal sections stained with hematoxylin and eosin at 10× magnification (scale bar 50 μm). (**A**) Pathological histology of sciatic nerve tissue on day 7 post-PSNL surgery. (**B**) Pathological histology of sciatic nerve tissue on day 14 post-PSNL surgery. (**C**) Swelling of nerve fiber on 7 and 14-day post-PSNL surgery. (**D**) Degenerated myelin sheath on 7 and 14-day post-PSNL surgery. (**E**) Hypertrophy of Schwann cells on 7 and 14-day post-PSNL surgery. (**F**) Necrosis on 7 and 14-day post-PSNL surgery. Black arrowhead shows swelling of nerve fiber, red arrowhead shows degenerated myelin sheath, blue arrowhead shows hypertrophy of Schwann cells and green arrowhead shows necrosis. The data is presented as the mean (n = 3) ± S.D. Different letters meant statistically significant differences: (***) *p* < 0.001 indicate significant differences from the PSNL control group. (###) indicate significant differences from the normal control group. *** *p* < 0.001 (two-way ANOVA followed by Dunnett’s test).

**Figure 6:**
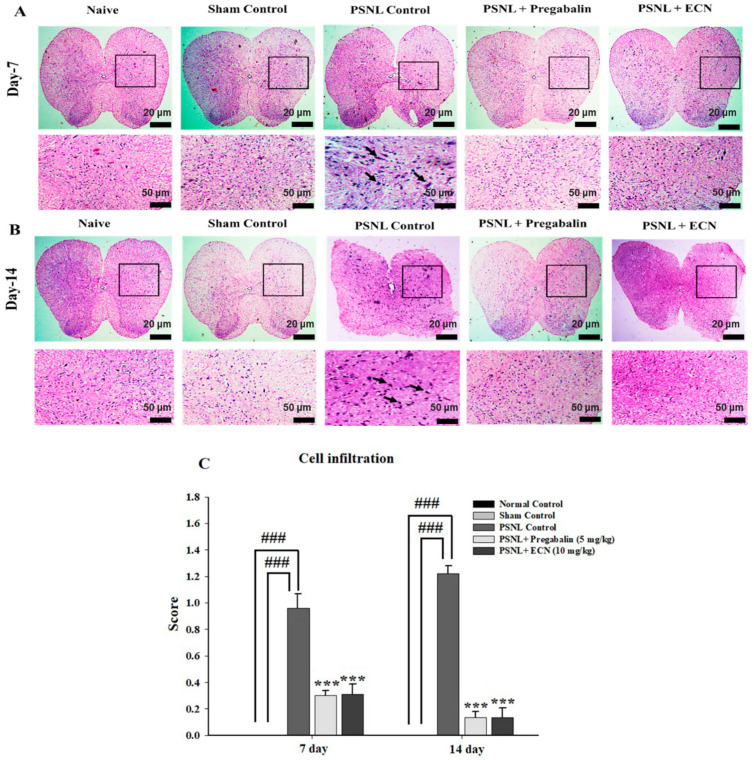
Effect of ECN (10 mg/kg) on the pathological histology of lumbar spinal cord tissue, the lumbar spinal cord of mice subjected to PSNL is shown in transverse sections stained with hematoxylin and eosin at 4× & 10× magnification (scale bar 20 μm or 50 μm). (**A**) Pathological histology of lumbar spinal cord tissue on day 7 post-PSNL surgery. (**B**) Pathological histology of lumbar spinal cord tissue on day 14 post-PSNL surgery. (**C**) Cell infiltration on 7 and 14-day post-PSNL surgery. The data is presented as the mean (n = 3) ± S.D. Different letters meant statistically significant differences: (***) *p* < 0.001 indicate significant differences from the PSNL control group. (###) indicate significant differences from the normal control group. *** *p* < 0.001 (two-way ANOVA followed by Dunnett’s test).

**Figure 7:**
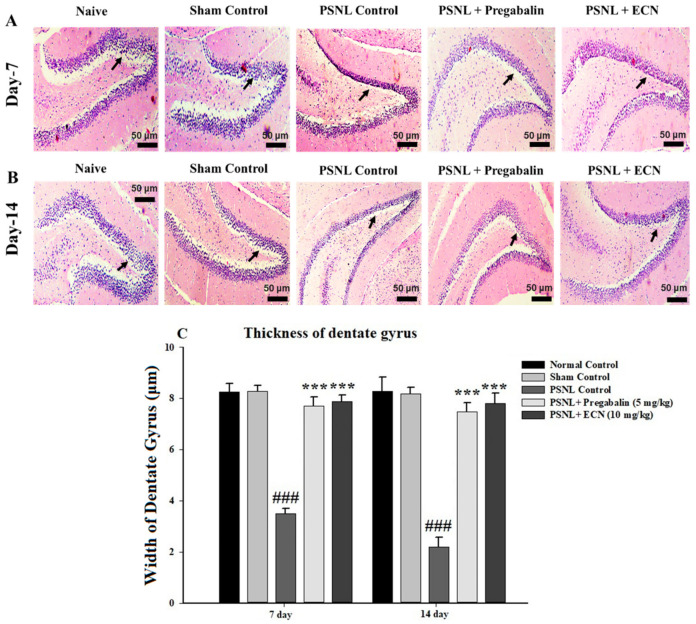
Effect of ECN (10 mg/kg) on the pathological histology of brain tissue, the dentate gyrus of mice subjected to PSNL is shown in coronal sections stained with hematoxylin and eosin at 10× magnification (scale bar 50 μm). (**A**) Pathological histology of brain tissue on day 7 post-PSNL surgery. (**B**) Pathological histology of brain tissue on day 14 post-PSNL surgery. (**C**) The thickness of dentate gyrus in micrometer (μm) on 7 and 14-day post-PSNL surgery. Black arrow heads show the thickness of the dentate gyrus. The data is presented as the mean (n = 3) ± S.D. Different letters meant statistically significant differences: (***) *p*< 0.001 indicate significant differences from the PSNL control group. (###) indicate significant differences from the normal control group. *** *p* < 0.001 (two-way ANOVA followed by Dunnett’s test).

H&E staining analysis of the sciatic nerve section showed that ECN (10 mg/kg) reduced the swelling of nerve fiber, degenerated myelin sheath, hypertrophy of Schwann cells and necrosis at days 7 and 14 post-PSNL surgery when observed under a light microscope (Figure 5). The myelin swelling was quantified in the longitudinal section of the sciatic nerve by using the Myelin J software as previously reported protocol [53,54]. Moreover, the ImagePro Plus software (Media Cybernetics, Silver Spring, MD, USA) was used for the quantification of necrosis according to the previously reported protocol [55]. ECN treated groups showed a reduction in cell infiltration in the transverse section of the lumbar spinal cord section at days 7 and 14 post-PSNL surgery in contrast to PSNL control when observed under a light microscope (Figure 6). Similarly, Myelin J software was used to quantify myelin swelling in the longitudinal section of the spinal cord and ImagePro Plus software (Media Cybernetics) was used for the quantification of necrosis and cell infiltration. Histopathological analysis of the coronal section of the brain showed a significant (*p* < 0.001) increase in the thickness of dentate gyrus in treatment groups when quantified by Image-J software 1.48 version (National Institutes of Health, Bethesda, MD, USA) (Figure 7).

### 2.6. Effect of ECN on Inflammatory Cytokines Expression

**Figure 8:**
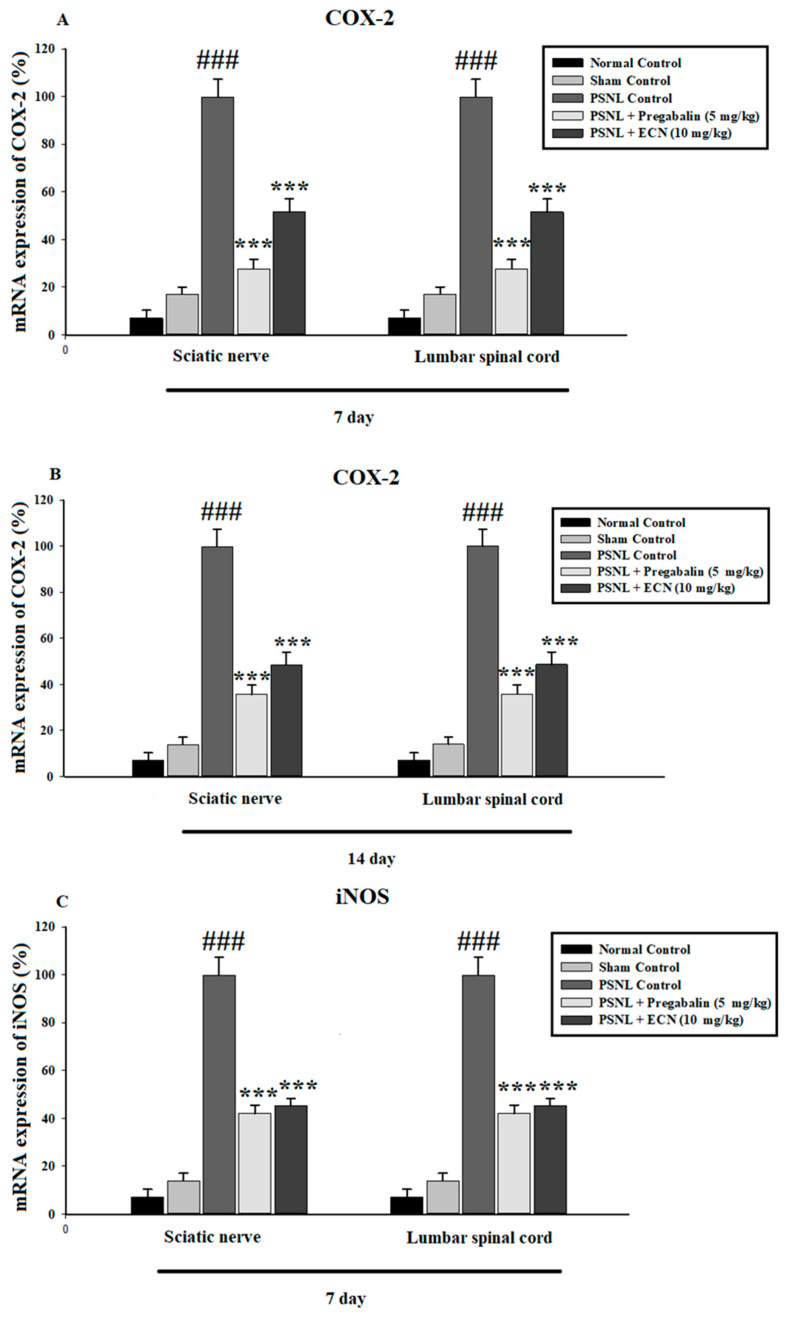

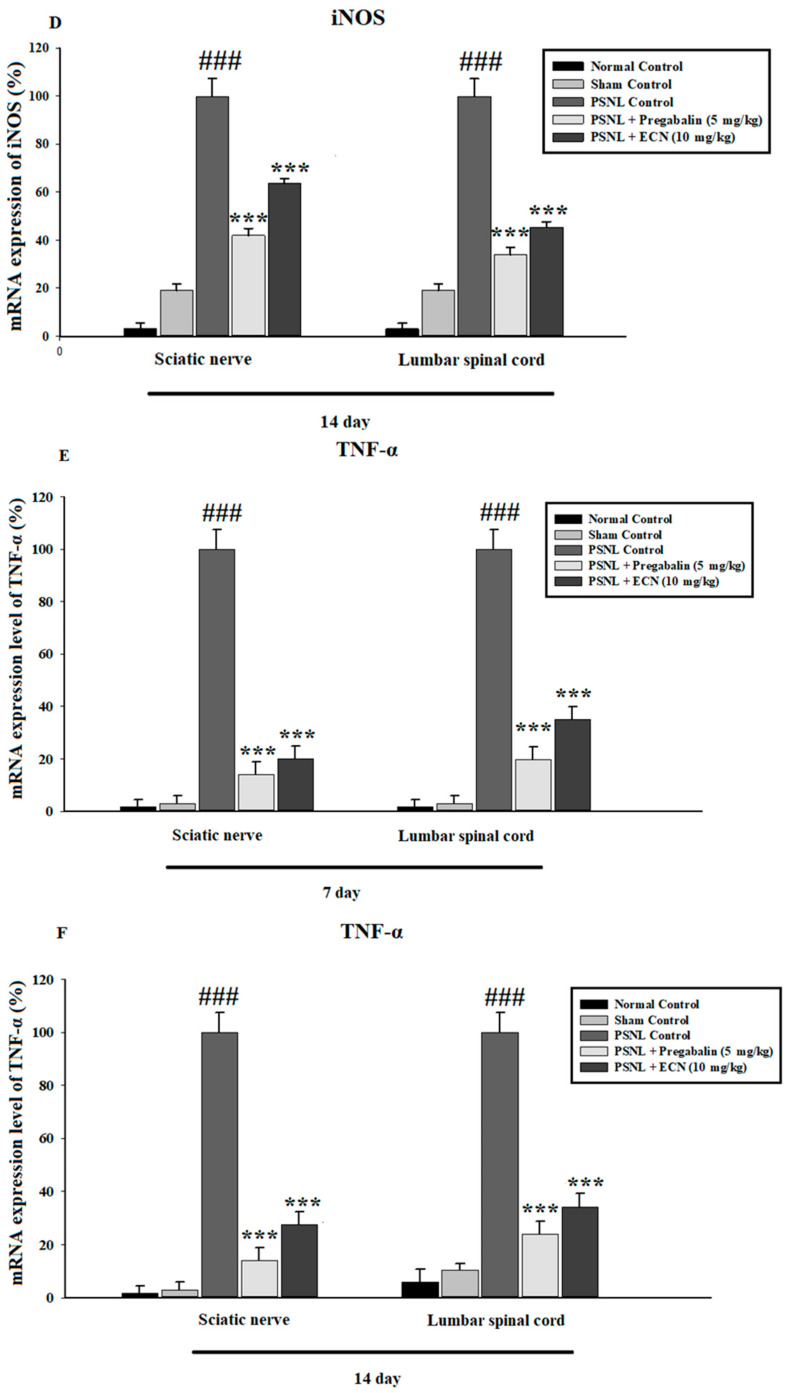

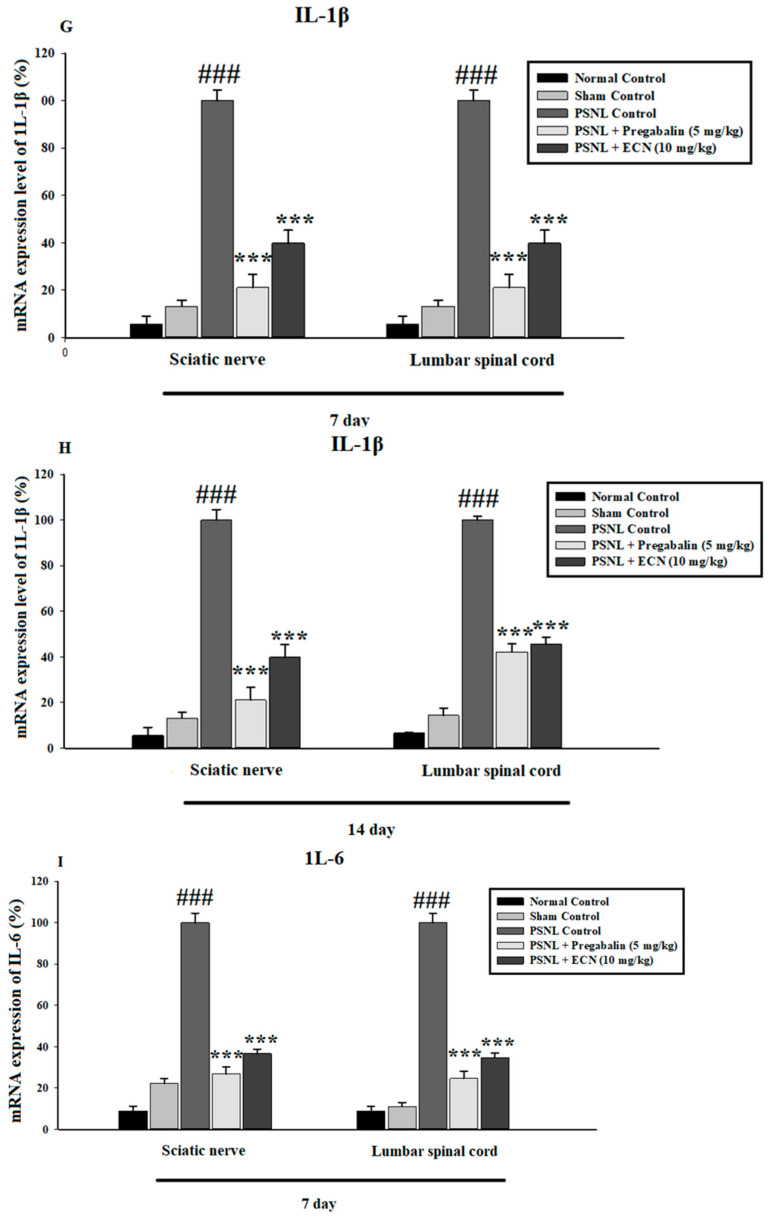

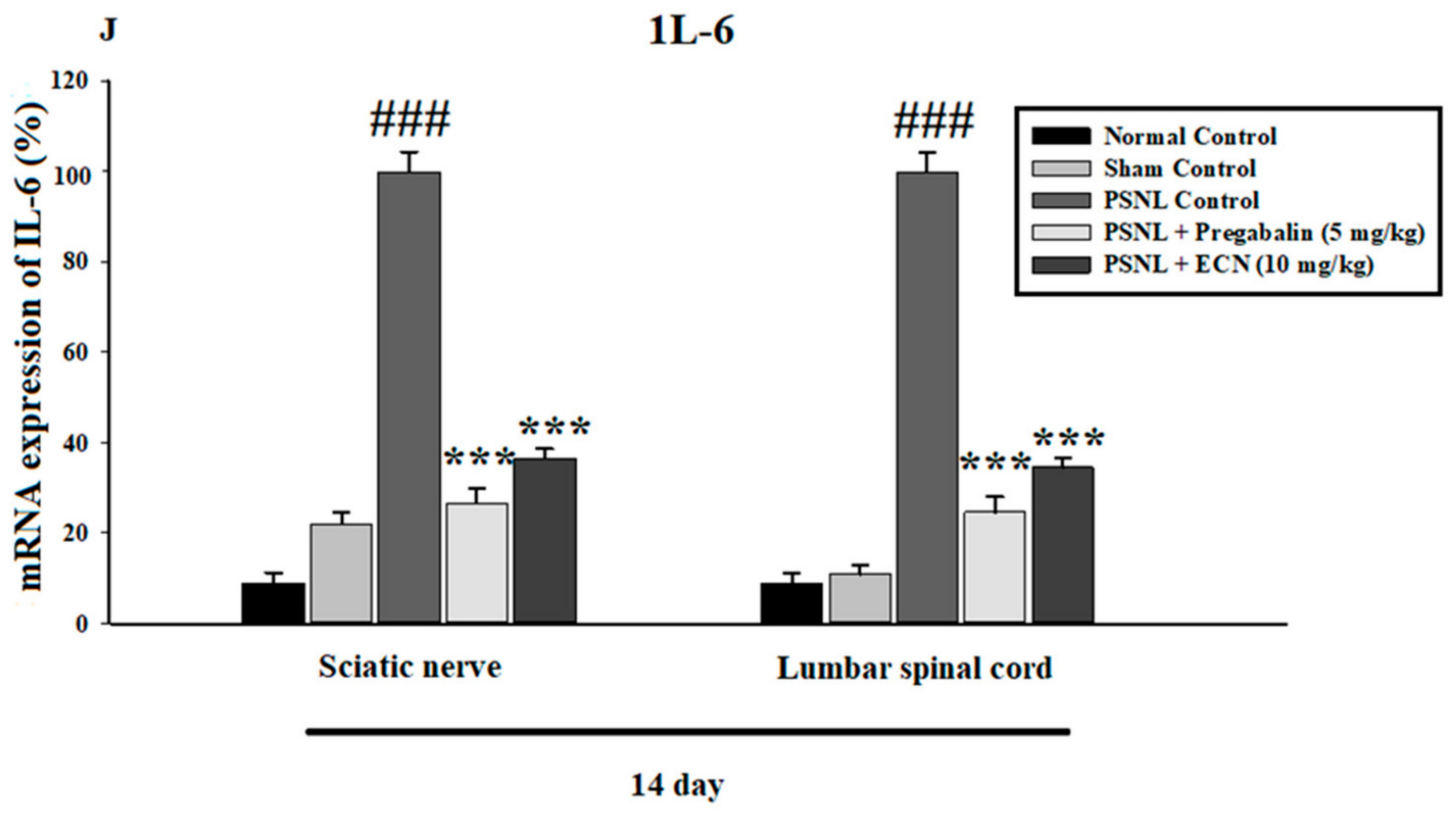
Effect of ECN (10 mg/kg) on the COX-2, iNOS, TNFα, IL-1β and IL-6 expression in the spinal cord and sciatic nerve tissue using PCR. (**A**) ECN (10 mg/kg) significantly inhibited the COX-2 in the sciatic nerve and spinal cord tissue on day 7 post-PSNL surgery. (**B**) ECN (10 mg/kg) significantly inhibited the COX-2 in the sciatic nerve and spinal cord tissue on day 14 post-PSNL surgery. (**C**) ECN (10 mg/kg) significantly inhibited the iNOS in the sciatic nerve and spinal cord tissue on day 7 post-PSNL surgery. (**D**) ECN (10 mg/kg) significantly inhibited the iNOS in the sciatic nerve and spinal cord tissue on day 14 post-PSNL surgery. (**E**) ECN (10 mg/kg) significantly inhibited the TNF-α in the sciatic nerve and spinal cord tissue on day 7 post-PSNL surgery. (**F**) ECN (10 mg/kg) significantly inhibited the TNF-α in sciatic nerve and spinal cord tissue on day 14 post-PSNL surgery. (**G**) ECN (10 mg/kg) significantly inhibited the IL-1β in the sciatic nerve and spinal cord tissue on day 7 post-PSNL surgery. (**H**) ECN (10 mg/kg) significantly inhibited the IL-1β in the sciatic nerve and spinal cord tissue on day 14 post-PSNL surgery. (**I**) ECN (10 mg/kg) significantly inhibited the IL-6 in sciatic nerve and spinal cord tissue on day 7 post-PSNL surgery. (**J**) ECN (10 mg/kg) significantly inhibited the IL-6 in sciatic nerve and spinal cord tissue on day 14 post-PSNL surgery. The data is presented as the mean (n = 3) ± S.D. Different letters meant statistically significant differences: (***) *p* < 0.001 indicate significant differences from the PSNL control group. (###) indicate significant differences from the normal control group. *** *p* < 0.001 (two-way ANOVA followed by Dunnett’s test).

The effect of ECN (10 mg/kg) was also investigated against various pro-inflammatory cytokines such as TNFα, IL-1β, IL-6, COX-2 and iNOS in the spinal cord and sciatic nerve tissues using quantitative RT-PCR. Neuropathic pain strongly induced expression of TNF-α, IL-1β, IL-6, COX-2 and iNOS, the treatment with ECN significantly decreased the mRNA expression levels of these inflammatory cytokines including TNF-α (F = 167.8, *p*< 0.001), IL-1β (F = 143.4, *p* < 0.001), IL-6 (F = 151.6, *p* < 0.001), COX-2 (F = 124.5, *p* < 0.001), and iNOS (F = 97.8, *p* < 0.001) as compared to the PSNL-treated group (Figure 8).

### 2.7. Effect of ECN on Anti-Oxidants Expression

**Figure 9:**
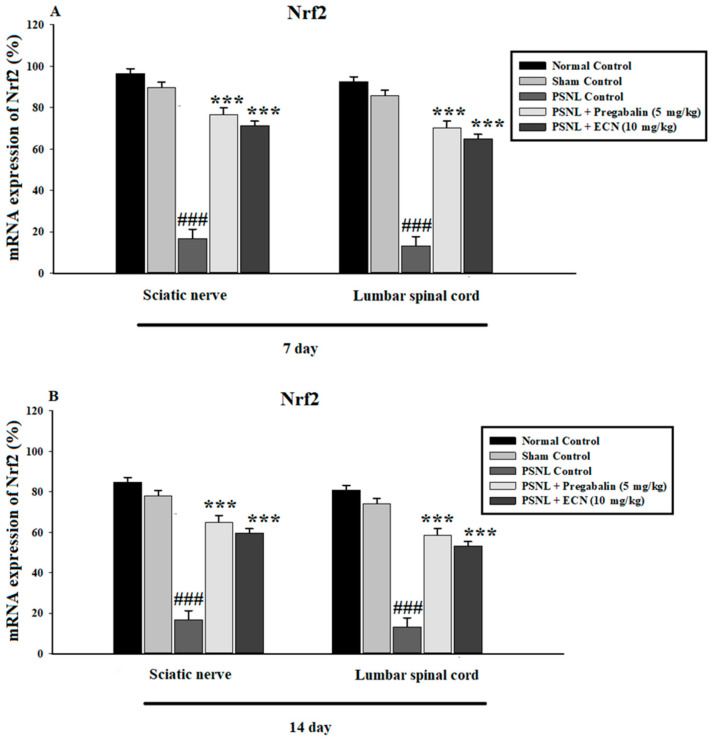

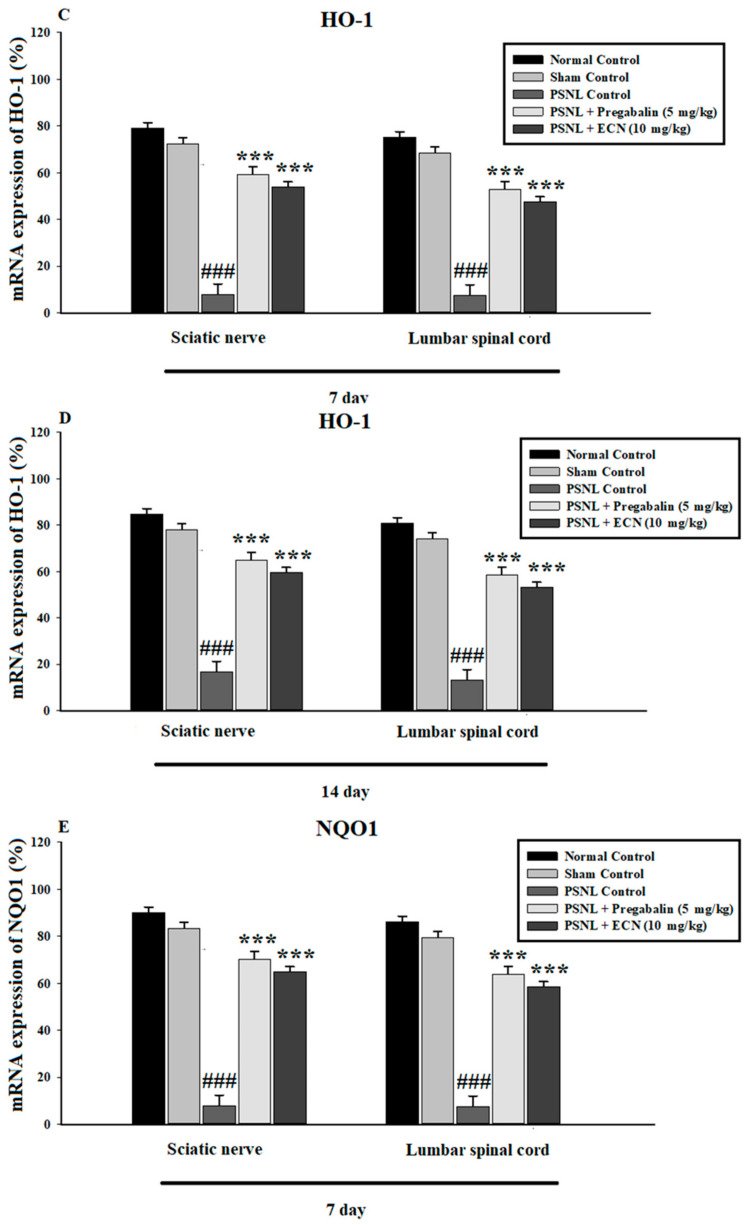

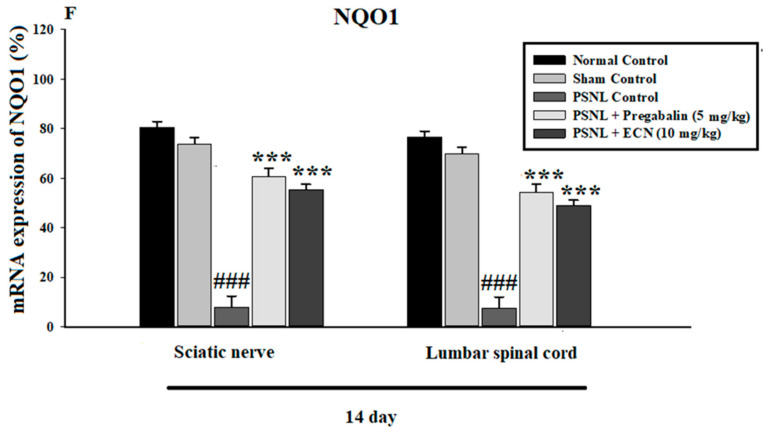
Effects of ECN (10 mg/kg, i.p.) on the oxidative stress markers in the spinal cord and sciatic nerve tissue using qtRT-PCR. (**A**) ECN (10 mg/kg, i.p.) treatment significantly enhanced the mRNA expression levels of Nrf2 in the sciatic nerve and spinal cord tissue on day 7 post-PSNL surgery. (**B**) ECN (10 mg/kg, i.p) treatment significantly enhanced the mRNA expression levels of Nrf2 in sciatic nerve and spinal cord tissue on day 14 post-PSNL surgery. (**C**) ECN (10 mg/kg, i.p) treatment significantly enhanced the mRNA expression levels of HO-1 in sciatic nerve and spinal cord tissue on day 7 post-PSNL surgery. (**D**) ECN (10 mg/kg, i.p) treatment significantly enhanced the mRNA expression levels of HO-1 in the sciatic nerve and spinal cord tissue on day 14 post-PSNL surgery. (**E**) ECN (10 mg/kg, i.p) treatment significantly enhanced the mRNA expression levels of NQO1 in sciatic nerve and spinal cord tissue on day 7 post-PSNL surgery. (**F**) ECN (10 mg/kg, i.p.) treatment significantly enhanced the mRNA expression levels of NQO1 in sciatic nerve and spinal cord tissue on day 14 post-PSNL surgery. The data is presented as the mean (n = 3) ± S.D. Different letters meant statistically significant differences: (***) *p* < 0.001 indicate significant differences from the PSNL control group. (###) indicate significant differences from the normal control group. *** *p* < 0.001 (two-way ANOVA followed by Dunnett’s test).

The mRNA expression levels of antioxidant enzymes and proteins such as Nrf2, HO-1 and NQO1 in the spinal cord and sciatic nerve of mice were determined using quantitative RT-PCR. Oxidative stress leads to the activation of reactive oxygen species which as a result amplifies the pathogenesis of neuropathic pain. The present results showed that there was a significant up-regulation of Nrf2 (F = 185.6, *p* < 0.001), HO-1(F = 177.4, *p* < 0.001) and NQO1 (F = 180.2, *p* < 0.001) enzyme due to ECN and pregabalin treatment as compared to the PSNL-treated group (Figure 9).

### 2.8. Effect of ECN on MDA Production

The MDA level in the sciatic nerve, spinal cord and brain (prefrontal cortex and hippocampus) was determined 7, 10 and 14 days after PSNL surgery. The upregulation of oxidative stress markers after nerve injury plays a major role in promoting the pathogenesis of neuropathic pain. Treatment with ECN and pregabalin significantly (*p* < 0.001) decreased the MDA level in prefrontal cortex (F = 208.7, *p* < 0.001), hippocampus (F = 219.6, *p* < 0.001) sciatic nerve (F = 215.5, *p* < 0.001), and spinal cord (F = 229.3, *p* < 0.001) as compared to PSNL control group (Figure S1).

### 2.9. Effect of ECN on NO Production

Nitrite level was determined in the brain (hippocampus, prefrontal cortex), spinal cord, and sciatic nerve tissue 7, 10- and 14-days post PSNL surgery (Figure S2). Excess reactive oxygen species lead to the increased production of NO which further worsens the neuropathic pain. Treatment with ECN significantly (*p* < 0.001) reduced the PSNL induced nitrite production in the prefrontal cortex (F = 198.5, *p* < 0.001), hippocampus (F = 225.8, *p* < 0.001), sciatic nerve (F = 251.2, *p* < 0.001), and spinal cord (F = 245.6, *p* < 0.001) as compared to PSNL control group (Figure S2), hence this confirmed the anti-inflammatory effect of drug.

### 2.10. Effect of ECN on SOD

SOD level was determined in brain (hippocampus, prefrontal cortex), spinal cord and sciatic nerve tissue 7, 10- and 14-days post PSNL surgery (Figure S3). Increased expression of antioxidant proteins and enzymes can treat the neuropathic pain induced by oxidative stress. Treatment with ECN and pregabalin significantly (*p* < 0.001) increased the level of SOD antioxidant enzyme in the prefrontal cortex (F = 188.3, *p* < 0.001), hippocampus (F = 175.4, *p* < 0.001), sciatic nerve (F = 170.7, *p* < 0.001), and spinal cord (F = 183.5, *p* < 0.001) as compared to PSNL control group (Figure S3), therefore this confirmed the antioxidant effect of ECN.

### 2.11. Effect of ECN on GSH

GSH level was determined in hippocampus, prefrontal cortex, lumbar spinal and sciatic nerve on 7, 10- and 14-day post PSNL surgery (Figure S4). Upregulation of antioxidant proteins and enzymes can treat the neuropathic pain induced by oxidative stress. Treatment with ECN and positive control significantly (*p* < 0.001) increased the level of GSH antioxidant protein in the prefrontal cortex (F = 167.5, *p* < 0.001), hippocampus (F = 155.4, *p* < 0.001), sciatic nerve (F = 149.7, *p* < 0.001), and spinal cord (F = 170.6, *p* < 0.001) as compared to PSNL control group (Figure S4).

### 2.12. Effect of ECN on GST

The GST level in the sciatic nerve, spinal cord and brain (prefrontal cortex and hippocampus) was determined on 7, 10- and 14-day after PSNL surgery (Figure S5). Neuropathic pain causes a significant increase in free radicals which as a result amplifies the pathogenesis of neuropathic pain. Treatment with ECN and pregabalin significantly (*p* < 0.001) increased the level of GST antioxidant enzyme in the prefrontal cortex (F = 196.7, *p* < 0.001), hippocampus (F = 217.4, *p* < 0.001), sciatic nerve (F = 189.5, *p* < 0.001), and spinal cord (F = 203.9, *p* < 0.001) as compared to PSNL control group (Figure S5).

### 2.13. Effect of ECN on Catalase

The catalase level in the brain (prefrontal cortex and hippocampus), spinal cord, and sciatic nerve was determined on 7, 10- and 14-day after PSNL surgery (Figure S6). Increased expression of antioxidant proteins and enzymes can treat the neuropathic pain induced by oxidative stress. Treatment with ECN and pregabalin significantly (*p* < 0.001) increased the level of catalase in the prefrontal cortex (F = 211.2, *p* < 0.001), hippocampus (F = 220.5, *p* < 0.001), sciatic nerve (F = 230.3, *p* < 0.001), and spinal cord (F = 225.9, *p* < 0.001) as compared to PSNL control group (Figure S6).

### 2.14. Effect of PSNL on Kidney and Liver Functions

To investigate whether sciatic nerve injury altered liver and kidney function, the AST, ALT, and creatinine levels were measured in blood plasma. The results demonstrated that sciatic nerve injury did not change plasma levels of AST and ALT and creatinine compared to the control group (Table 1). To further strengthen our data, we examined the histopathology of the liver and kidney tissue. Histopathology results revealed that the photomicrograph of the PSNL group shows normal histology with no histopathological alteration (Figure S7).

### 2.15. Effect of ECN on Nrf2 and Caspase-3 Expression

**Figure 10:**
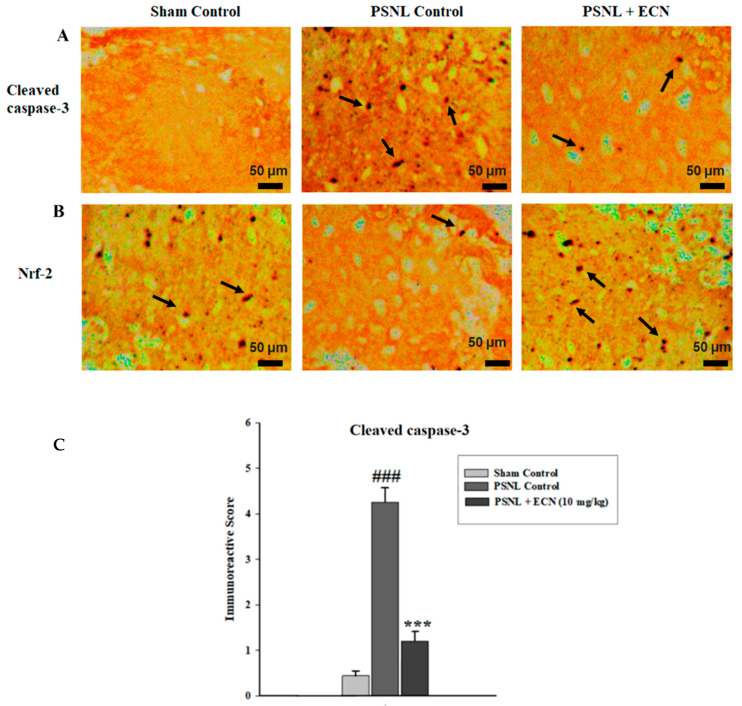

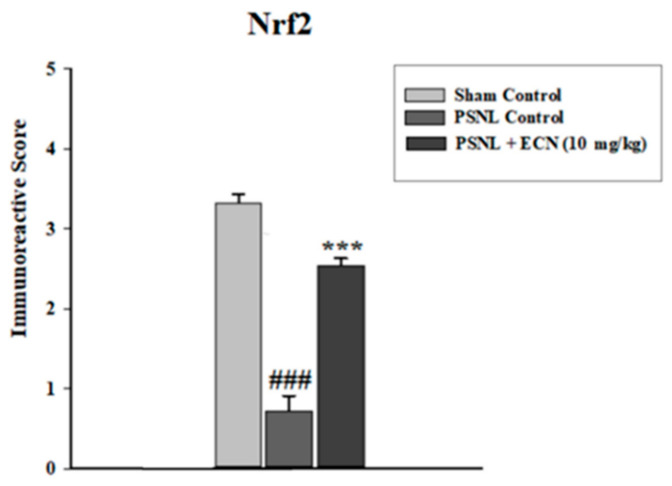
Effect of ECN (10 mg/kg, i.p) on the immunohistochemical staining of mice spinal cord, showing the effect of ECN on PSNL induced expression of caspase-3 (**A**) and Nrf2 (**B**). ECN treatment reduced the expression of caspase-3 and enhanced the expression of Nrf2 (**C**). Black arrow heads show the expression of caspase-3 and Nrf2 in the spinal cord. (10× magnification, scale bar 50 μm). The data is presented as the mean (n = 3) ± S.D. Different letters meant statistically significant differences: (***) *p* < 0.001 indicate significant differences from the PSNL control group. (###) indicate significant differences from the normal control group. *** *p* < 0.001 (one-way ANOVA followed by Bonferroni post-test).

The modulatory action of ECN was further strengthened by assessing the expressions of caspase-3 and Nrf2 by using immunohistochemistry. Caspase-3, a pro-apoptotic protein plays a vital role in the pathogenesis of neuropathic pain. The present result showed that ECN post-treatment significantly decreased (F = 130.5, *p* < 0.001) the immune-labelling of caspase-3 as compared to the PSNL control (Figure 10A,C). The underlying cause of oxidative stress-mediated neuropathic pain is the under-expression of Nrf2, a transcription factor involved in the regulation of genes that encodes antioxidant proteins and phase 2 detoxifying enzymes. ECN post-treatment significantly (F = 109.7, *p* < 0.001) upregulated the expression of Nrf2 as compared to the PSNL control when observed under a light microscope. In the present study, the Image-J software 1.48 version was used to measure the relative expression of caspase-3 and Nrf2 according to previously reported protocols [56,57] (Figure 10B,C).

### 2.16. Effect of ECN on Myelin Sheath of Sciatic Nerve

**Figure 11:**
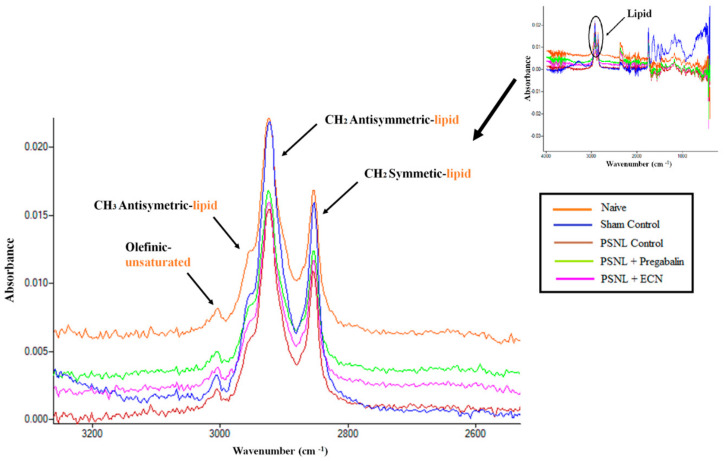
Effects of ECN (10 mg/kg) on the lipid content of the sciatic nerve. The FTIR spectra of the naive, sham control, PSNL control, PSNL + pregabalin and PSNL + ECN treated sciatic nerve in the range of 3025–2800 cm^−1^.

ECN exhibited a protective effect on the lipid content of the myelin sheath which shows its potential to treat nerve damage or neuropathy (Figure 11). The change in unsaturated fatty acid lipid content is determined from the analysis of the olefinic band, arisen from HC=CH groups [58]. The wavenumber of this band was shifted significantly (*p* < 0.001) to a higher value in the ECN treated group as compared to the PSNL control group (Table 2). The CH_2_ antisymmetric (2927 cm^−1^), and the CH_2_ symmetric (2857 cm^−1^) stretching bands originates mainly from lipids. The CH_3_ antisymmetric (2959 cm^−1^) has an equal contribution from lipids and proteins (Table 3) [59,60,61,62]. The wavenumber of these bands in the ECN group was shifted significantly (*p* < 0.001) to a higher value as compared to the PSNL control group (Table 2).

## 3. Discussion

Chronic pain initiated by a primary lesion or dysfunction in the nervous system and leads to prolonged changes in pain pathway structures (neuroplasticity) and abnormal processing of sensory information [65]. Chronic pain is a big challenge to the healthcare scientist [66]. PSNL model produces unilateral peripheral mononeurotherapy as observed in humans that can be modeled for causalgia (incessant burning pain) and regional pain syndrome in rodents. Numerous drugs are already in the market for the treatment of neuropathic pain [65,67]. The drugs for the treatment of neuropathic pain exhibited severe and serious side effects [65]. Therefore, there is a need to seek out new drugs with more safety and more efficacious profile.

The present study explores the potential effects of naturally isolated ECN in the PSNL-induced model of neuropathic pain. Intraperitoneal administration of ECN (10 mg/kg) post-treatment once daily starting from day 3 after surgery, significantly attenuated mechanical hyperalgesia, thermal hyperalgesia, mechanical allodynia and cold allodynia. A decreased score of distress symptoms such as general health, changes in temperament, gait weakness and reluctance to move were observed in the treatment group as compared to the PSNL control group. In addition, ECN (10 mg/kg) intraperitoneal administration from day 3 after surgery reversed histopathological abnormalities of PSNL-induced neuropathic pain. Our findings suggest that ECN had no disturbing effect on muscle coordination and motor activity.

Earlier studies have reported the activation of pro-inflammatory cytokines and apoptotic markers in the induction and maintenance of neuropathic pain [68,69]. The upregulation of inflammation and apoptotic proteins after injury plays a crucial role in aggravating the neuropathic pain [70]. Previous findings reported that the up-regulation of inflammatory and apoptotic markers after nerve injury plays a major role in promoting the pathogenesis of neuropathic pain [71,72]. In pathological conditions, excess reactive oxygen species and apoptosis leads to the increased production of pro-inflammatory cytokines which further worsens the neuropathic pain [73]. In the present study we found that pro-inflammatory (TNF-α, IL-1β and IL-6) and pro-apoptotic (caspase-3) proteins were elevated in the spinal cord and sciatic nerve of mice on day 7 and 14 after PSNL surgery. Treatment with ECN decreased the TNF-α, IL-1β, IL-6 and caspase 3 expression in contrast to the PSNL group.

Recent findings suggest that iNOS releases NO and subsequently peroxynitrite which could lead to the production of pro-inflammatory cytokines and could participate in neuropathic pain [74]. Moreover, it has been demonstrated that increased production of iNOS and TNF-α could be responsible for the development of neuropathic pain [71]. In pathological conditions, large amounts of pro-inflammatory cytokines such as iNOS and TNF-α are released which further causes the production of reactive oxygen species (ROS) [36]. In the current study, ECN showed an anti-inflammatory effect by inhibiting the expression of iNOS and TNF-α.

Nrf2, one of the basic leucine zipper transcription factor that can bind to ARE-sequence is important for protection against oxidative stress [75]. Many chronic neurological disorders such as multiple sclerosis and neuropathic pain are thought to involve oxidative stress as a factor contributing to the progression of the disease [76]. Earlier studies reported that activation of the Nrf2 pathway might be a contributing factor for the treatment of neuropathic pain [18]. It has been found that Nrf2 gene expression is vital for the maintenance and responsiveness of the cell’s defense systems [36]. In the current study, we found that increased Nrf2 gene expression can treat the neuropathic pain induced by oxidative stress. Treatment with ECN increased the expression of Nrf2 and its downstream proteins such as HO-1 and NQO1 in treatment groups as compared to the PSNL group.

Previous studies have found that activation of apoptosis might be involved in the pathogenesis of neuropathic pain [36]. Apoptotic pathways lead to the activation of caspase-3 which leads to cell death [77]. Caspase-3, a pro-apoptotic protein plays a vital role in the pathogenesis of neuropathic pain [78]. Moreover, it has been demonstrated that inhibition of caspase 3 would inhibit apoptosis and thermal hyperalgesia following chronic constriction injury [79]. In this study, caspase-3 expression in the treatment group was reduced as compared to the PSNL control group. This shows the protective effect of ECN in neuropathic pain.

Quantitative DNA damage in sciatic and spinal cord tissues was analyzed with the help of comet assay. DNA disruption was identified by DNA relocation out of the nucleus and into the tail of the comet. DNA strand breakage can be due to the enhanced generation of ROS leading to free radical formation which might be the reason for the alteration and breakage of double-helical strands causing cell death [80]. This DNA disruption was noticeably attenuated by ECN representing its neuroprotective potential. Moreover, the coronal section of the brain tissue, longitudinal section of the sciatic nerve and longitudinal section of the lumbar spinal cord were stained with H&E and then observed under a microscope. It was found that the dentate gyrus of the hippocampus in the PSNL-challenged group was reduced in thickness as compared to the treatment group. In the same way, increased signs of inflammation were observed in PSNL-induced sciatic nerve and lumbar spinal cord demonstrating the debilitating condition of the PSNL control group. Although, such a pattern was not seen in the treatment group. This shows the efficient potential of ECN in treating peripheral neuropathy.

To elucidate the lipid content of myelin sheath of the sciatic nerve in the PSNL model, FTIR spectroscopy was used in this study since it enables efficient, rapid and simultaneous monitoring of small changes in biochemical components and processes in diseases or drug-induced pathological conditions [58,61]. Damage to myelin sheath via alteration in the biochemical make-up of the sciatic nerve tissue contributes to neuropathy. ECN exhibited a protective effect on the lipid content of the myelin sheath which shows its potential to treat nerve damage or neuropathy. The change in unsaturated fatty acid lipid content is determined from the analysis of the olefinic band, arisen from HC=CH groups. In the current study, the wavenumber of this band was shifted significantly to a higher value in ECN treated groups as compared to the PSNL control group. Thus, the anti-neuropathic potential of ECN might be due to the alleviation of distress symptoms, biochemical alteration in sciatic nerve morphology, and modulation of expression of anti-oxidant proteins, pro-apoptotic proteins, and pro-inflammatory cytokines.

## 4. Materials and Methods

### 4.1. Plant Material

ECN was isolated from dried buds of *T. farfara* (mentioned compound was received from Prof. Yeong Shik Kim, Seoul National University, South Korea) and identified by comparison with spectral data (^1^H-NMR and ^13^C-NMR), as previously reported in the literature [81]. The voucher specimen number is EA333 deposited at Natural Product Chemistry Laboratory, College of Pharmacy, Ewha Womans University, Seoul, Korea. The extraction and purification of ECN was reported by our team elsewhere [52,82].

### 4.2. Chemicals and Reagents

Pregabalin was purchased from Sigma–Aldrich (Steinheim, Germany). Ketamine (Ketarol, 500 mg/10 mL) was purchased from Global Pharmaceutical (Islamabad, Pakistan). Pyodine was purchased from Brookes Pharma (Karachi, Pakistan). All the other chemicals used in the current study were of analytical grade. All the drugs described above were freshly prepared for the study, dissolved in 2% DMSO and diluted with 0.9% saline.

### 4.3. Animals and Surgical Procedure

Male albino mice (3–4 weeks of age) having weight of 25–30 g were purchased from the National Institute of Health (NIH), Islamabad, Pakistan. Animals were housed in a controlled laboratory environment at a temperature of 23 ± 1 °C in 50 ± 10% humidity under a 12 h light-dark cycle. Standard lab chow and water were provided throughout the experiment. All the experiments were performed in accordance with the Quaid-i-Azam University, Islamabad regulations governing the care and use of Laboratory Animals, and conformed to the ethical guidelines for the study of experimental pain in conscious animals established by the International Association for the Study of Pain. The current study was permitted by the Bioethical Committee of Quaid-i-Azam University, Islamabad (Permit No: BEC-FBS-QAU2018-125).

Peripheral neuropathic pain was induced in mice by PSNL as previously described [83]. Briefly, mice were deeply anesthetized with ketamine (100 mg/kg i.p.), the left sciatic nerve was exposed after the incision of skin and the exposed skin was swabbed with a povidone-iodine topical 10% *w*/*v* solution. The dorsal 1/3 to 1/2 of the sciatic nerve was tightly ligated with an 8-0 silk suture just distal to the point at which the posterior biceps-semitendinosus nerve branches off. After performing partial nerve ligation, muscle and skin layer were at once sutured with thread, and a topical antibiotic was applied. In sham-operated mice, an identical operation was performed, except that the sciatic nerve was not ligated. All surgical procedures were conducted under normal sterile conditions.

### 4.4. Drug Treatment and PSNL Model

**Figure 12:**
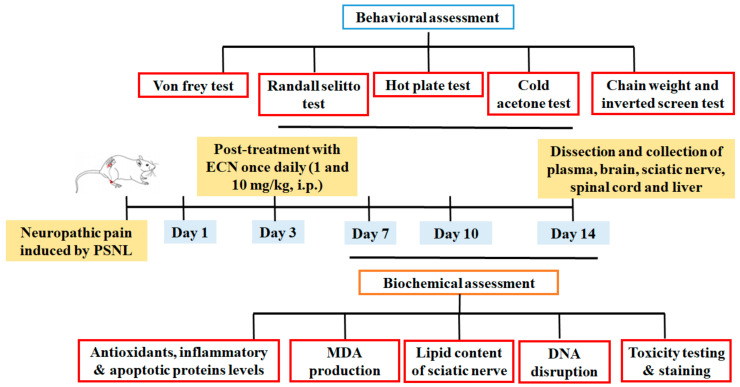
Experimental design for investigating the effect of ECN (1 and 10 mg/kg) against PSNL neuropathic pain.

Mice were randomly divided into six groups (n = 10/group). The sample size was selected according to the previously established protocol [84]. Optimal administration doses were selected according to the results of the preliminary experiments. ECN (1 and 10 mg/kg) was administered i.p. to neuropathic mice once a day for 11 days, starting from the third day after surgery (Figure 12). Positive control (pregabalin 5 mg/kg) was also administered i.p. to neuropathic mice once a day for 11 days, starting from the third day after surgery. No treatment was given to normal, sham and PSNL control groups. Three to four mice from each group were sacrificed on days 7, 10 and 14 after PSNL surgery for biochemical evaluations (Figure 12).

Group 1: Normal (naïve mice; did not undergo any surgical procedure)
Group 2: Sham control (sciatic nerve exposure without nerve ligation)
Group 3: PSNL control (sciatic nerve exposure with nerve ligation)
Group 4: PSNL+ pregabalin (5 mg/kg) (sciatic exposure with nerve ligation and pregabalin (5 mg/kg) administered)
Group 5: PSNL+ECN (1 mg/kg) (sciatic exposure with nerve ligation and ECN (mg/kg) administered)
Group 6: PSNL+ECN (10 mg/kg) (sciatic exposure with nerve ligation and ECN (10 mg/kg) administered)

### 4.5. Behavior Test

The experimental animals were subjected to various behavioral studies for investigation of mechanical hyperalgesia, thermal hyperalgesia, mechanical allodynia, cold allodynia, and muscle coordination carried out on the different time intervals of 1 day before PSNL surgery and 3, 7, 10, and 14 days post-PSNL surgery. The responses to mechanical hyperalgesia, thermal hyperalgesia, mechanical allodynia, cold allodynia, muscle, and motor coordination were measured before, and 3, 7, 10, and 14 days after surgery (24 h after the last administration of ECN or positive control).

#### 4.5.1. Distress Symptoms and Survival Rate

The number of mice surviving in each group and the mortality rate was recorded daily as previously described [85,86]. Animals were observed throughout the experiment to record distress symptoms. Distress symptoms included general health, changes in temperament, gait weakness and reluctance to move. Scoring was carried out according to the criteria mentioned previously [85].

#### 4.5.2. Mechanical Hyperalgesia

To monitor the mechanical hyperalgesia, Randall Sellito (Digital Paw Pressure Randall Selitto Meter, IITC Life Science Inc. Wood land Hills, CA, USA) was used as per the method previously described with slight modifications [87,88]. Before the start of the test, the mice were placed in a quiet room for 15–30 min to acclimatise to the environment. This test consists of evoking a hind paw flexion reflex with a hand hold force transducer and the force exerted is noted on the screen. The tip of the Randall Sellito was applied perpendicular to the plantar surface of the ipsilateral left hind paw and the pressure was increased gradually. The final reading of the Randall Sellito is the characteristic withdrawal of the paw and clear movement of the mice. The duration of paw withdrawal (PWD) was recorded with a cut-off latency of 15 s [89]. Mechanical hyperalgesia was evaluated before and after the initiation of the treatment. Three consecutive readings were taken for statistical analysis. The test was performed 1 day before PSNL surgery and 3, 7, 10- and 14-days post PSNL surgery.

#### 4.5.3. Thermal Hyperalgesia

Thermal hyperalgesia of the plantar surface of the ipsilateral left hind paw was assessed as described previously [90,91]. The temperature of the hot plate was kept at 50 ± 0.5 °C. Once the animals were placed on the top of the preheated hot plate, the paw licking was taken as a positive response. The paw withdrawal latency (PWL) and PWD were recorded with a cut-off latency of 35 s. Thermal hyperalgesia was evaluated before and after the initiation of the treatment. Three consecutive readings were taken for statistical analysis. The test was performed 1 day before PSNL surgery and 3, 7, 10- and 14-days post PSNL surgery.

#### 4.5.4. Mechanical Allodynia

In order to monitor the mechanical allodynia, a series of 9 von Frey filaments (0.4, 0.6, 1, 1.4, 1.8, 2, 4, 6 and 8 g) were used as per the method previously described with slight modifications [92,93]. The filaments were applied to the plantar surface of the ipsilateral left hind paw and the applied force was increased gradually. Lifting or licking the paw was considered as a withdrawal response and the time taken to show a withdrawal response (PWL) three out of five times was considered positive. A cut-off latency of 15 s was imposed [89]. Three consecutive readings were taken for statistical analysis. The test was performed 1 day before PSNL surgery and 3, 7, 10- and 14-days post PSNL surgery.

#### 4.5.5. Cold Allodynia

In order to assess cold allodynia, the acetone drop method was employed as per the method previously described with slight modifications [94,95]. A 25 µL volume of acetone was sprayed onto the mid-plantar surface of the ipsilateral left hind paw, using a blunt needle connected to a syringe without touching the paw. The duration of the withdrawal response (PWD) during 60 s of the acetone application to the plantar surface of the ipsilateral left hind paw was recorded. Three consecutive readings were taken for statistical analysis. The test was performed 1 day before PSNL surgery and 3, 7, 10- and 14-days post PSNL surgery.

#### 4.5.6. Muscle Coordination and Motor Coordination

In order to determine the sedative effect of ECN on muscle strength and motor coordination, Kodzeila’s inverted screen was performed as described previously [96]. Each mouse was placed in the center of the wire mesh screen and the screen was inverted over 2 s, with a mouse’s head declining first. Investigator was trained to hold the screen steadily 40–50 cm above a padded surface. Time was recorded using a digital stopwatch when the mouse falls off and the score was assigned according to the protocol [97]. A cut-off latency of 60 s was imposed [98]. Three consecutive readings were taken for statistical analysis. The test was performed 1 day before PSNL surgery and 3, 7, 10- and 14-days post PSNL surgery.

For a full assessment of motor deficit, weight lifting test was performed as described previously [99]. Each weight was prepared using a thin wire mesh to which a length of steel chain consisting of from 1 to 7 links, each weighing 13 g, was attached. Each mouse was held by the tail and successively allowed to grasp a series of increasing weights, with a rest of 10 s between each lift. A hold of 3 s is the criterion. Time was recorded using a digital stopwatch when the mice dropped the weight in less than 3 s. If it held the weight for 3 s then it was allowed to grasp the next heavier weight. The score was assigned to each mouse. Three consecutive readings were taken for statistical analysis. The test was performed 1 day before PSNL surgery and 3, 7, 10- and 14-days post PSNL surgery.

### 4.6. Biochemical Experiments

#### 4.6.1. Comet Assay

DNA damage in the sciatic nerve and spinal cord tissues were assessed following 14 days of post-PSNL surgery by comet assay as described previously [100,101]. Small pieces of the sciatic nerve and spinal cord collected from mice after sacrifice were suspended separately in 1 mL of cold lysing solution i.e., Ca^2+^ and Mg^2+^ free Hanks’ balanced salt solution (HBSS) in a microcentrifuge tube and then the tissues were homogenized separately. About 5–10 μL of the cell suspension was mixed in 0.5% low melting point agarose (LMPA), layered on the slides precoated with 1% normal agarose solution (NMA), and then covered with a coverslip, retained for 10 min on an ice pack. After repeating this step twice, the slides were placed in a lysing solution for 2 h at 4 °C. After electrophoresis slides were stained with 1% ethidium bromide and were examined under a fluorescent microscope. The amount of DNA damage was analysed by CASP 1.2.3.b software (Krzysztof Końca, CaspLab.com). The tail length and % DNA in the tail was used to assess the amount of DNA damage.

#### 4.6.2. Histopathological Analysis

Hematoxylin and eosin (H&E) staining of the sciatic nerve, lumbar spinal cord and brain tissues were performed following 7 and 14 days post-PSNL surgery. The fresh tissue samples were immediately stored in the fixative solution (10% formalin) overnight at 4 °C. On the second day, the tissue samples were dehydrated using graded alcohols and a xylene substitute. The tissue specimens were infiltrated and embedded in paraffin. The paraffin block was sectioned at 4-µm by microtome and was stained with H&E according to the protocol previously reported [102]. The stained sections were observed under the light microscope (100×).

#### 4.6.3. Quantitative Real-Time Reverse Transcriptase-Polymerase Chain Reaction

The quantitative RT-PCR was used to determine the effect of ECN (10 mg/kg) on the mRNA expression levels of inflammatory mediators (COX-2, iNOS, TNFα, IL-1β and IL-6) and anti-oxidant proteins (Nrf2, HO-1 and NQO1) following 7 and 14 days post-PSNL surgery. Trizol reagent was used to isolate the total RNA from the sciatic nerve and lumbar dorsal spinal cord (L4–L6) tissue of mice as described previously [103,104]. Briefly, GenDEPOT 0.2 mL 8-strip tubes were used for quantitative PCR. 10 uL each of forward primer and reverse primer along with 80 μL DEPC-treated water (Sigma-Aldrich) and the fluorescent dye, SYBR green working solution was used. The reaction conditions were as follows 95 °C for 5 min followed by 40 cycles at 95 °C for 1 min (denaturation), then 55 °C for 45 s, and lastly 72 °C for 30 s (annealing and elongation). The optimal conditions, melting point, and reaction specificity were determined beforehand. 7300 real-time PCR system software was used for analysis. Beta-actin, a housekeeping gene, was chosen as an internal standard.

#### 4.6.4. Determination of Malondialdehyde (MDA)

To investigate the effect of ECN (10 mg/kg. i.p.) on oxidative stress marker, MDA level was quantified in the sciatic nerve, lumbar dorsal spinal cord (L4–L6), hippocampus and prefrontal cortex tissues of day 7, 10 and 14 post PSNL surgery as described previously [105,106]. The concentration of MDA, a marker of lipid peroxidation was analysed in the form of thiobarbituric acid reacting proteins (TBARS).

#### 4.6.5. Determination of Nitric Oxide (NO)

NO level in the sciatic nerve, lumbar dorsal spinal cord (L4–L6), hippocampus and prefrontal cortex tissues of day 7, 10 and 14 post-PSNL surgery were determined by Griess assay according to the method previously described [90,107]. The concentration of NO was determined by using Griess reagent, 1% sulfanilamide and 2.5% phosphoric acid. The concentration of nitrite was determined by measuring absorbance at 540 nm.

#### 4.6.6. Determination of Superoxide Dismutase (SOD)

To investigate the effect of ECN (10 mg/kg. i.p.) on the anti-oxidant marker, SOD level was quantified in sciatic nerve, lumbar dorsal spinal cord (L4–L6), hippocampus and prefrontal cortex tissues of day 7, 10 and 14 post-PSNL surgery according to the method previously described [31,75]. The SOD activity was measured by taking Tris-EDTA buffer (50 Mm, pH 8.5), pyrogallol (24 mM) and 10 µL of the sample in a total volume of 0.2 mL. The final readings were noted in triplicate at 420 nm.

#### 4.6.7. Determination of Glutathione (GSH)

GSH level was quantified in the sciatic nerve, lumbar dorsal spinal cord (L4–L6), hippocampus, and prefrontal cortex tissues of day 7, 10, and 14 post-PSNL surgery as described previously [80]. The reduced glutathione level was measured by mixing 0.1 mL of tissue homogenate obtained from each dilution and 2.4 mL from the phosphate buffer solution. The final volume was adjusted to 3 mL by adding 0.5 mL DTNB solution. The absorbance was measured at 412 nm.

#### 4.6.8. Determination of GST

GST level was quantified in the sciatic nerve, lumbar dorsal spinal cord (L4–L6), hippocampus, and prefrontal cortex tissues of day 7, 10, and 14 post-PSNL surgery as described previously [87]. The enzyme was quantified by its ability to conjugate GSH and CDNB. The level of GST was determined by mixing 0.1 mL of homogenate and 0.1 mL of CDNB. Finally, 0.1 M phosphate (pH 6.5) buffer was added to make the final volume up to 3 mL. The absorbance was measured at 314 nm.

#### 4.6.9. Determination of Catalase (CAT)

Catalase level was quantitated in the sciatic nerve, lumbar dorsal spinal cord (L4–L6), hippocampus and prefrontal cortex tissues of day 7, 10, and 14 post-PSNL surgery according to previously described methodology [105]. The catalase activity was determined by taking 3 mL of H_2_O_2_-phosphate buffer in an experimental cuvette and by the rapid addition of 40 µL of an enzyme extract. The final readings were noted in triplicate at 240 nm.

#### 4.6.10. Analysis of Renal and Hepatotoxicity

The systemic toxicity associated with sciatic nerve injury was assessed by measuring the levels of aspartate aminotransferase (AST), alanine aminotransferase (ALT), and creatinine in the blood. The whole blood obtained 14 days after PSNL surgery was centrifuged at 5000 rpm for 5 min to separate the serum from blood cells and the temperature was maintained at 4 °C. The serum was used to determine the level of ALT, AST, and creatinine. Moreover, histopathological analysis such as H&E staining was also performed to analyzed histopathology of renal and hepatic tissue.

#### 4.6.11. Immunohistochemistry

Immunohistochemistry of the Nrf2 (antioxidant) and caspase-3 (apoptotic markers) was performed using the avidin-biotin-peroxidase complex (ABC) method to detect expressions of the mentioned markers in the paraffin-embedded sections of the mouse spinal cord according to previously reported protocol [100]. Briefly, the sections were deparaffinized in xylene and then graded hydrated in alcohol. Then, antigens were retrieved by the enzymatic method and then treated with PBS. Then, 3% H_2_O_2_ in methanol was used for blocking endogenous peroxidases for 10 min. Subsequently, incubation of these slides was done with normal goat serum (5%) containing 0.1% Triton X-100, followed by overnight incubation with rabbit antibodies (anti-Nrf2 and anti-caspase-3) (Santa Cruz Biotechnology, Dallas, TX, USA). The concentration of primary antibody was (1/1000), 0.5 µL of primary antibody in 500 µL blocking buffer (×3) → 1.5 µL of antibody/1500 µL of blocking buffer. This process continued for the next day. These slides were further incubated with biotinylated anti-rabbit secondary antibody (Santa Cruz Biotechnology) followed by washing with 0.1 M PBS solution. The concentration of secondary antibody was (1/500), 3 µL of secondary antibody in 1500 µL blocking buffer (×3) → 9 µL of antibody/4.5 mL of blocking buffer. The incubation process continued, next with the ABC Elite Kit in a humidified chamber for 1 h. Then, 0.1 M PBS was again used for the washing of slides. Each marker expression was labeled with peroxidase and colored with diaminobenzidine (DAB) for the detection of the antigen-antibody complex. Further, slides were again washed in distilled water and graded ethanol dilutions were finally used for dehydration. These slides were fixed in xylene and coverslips were placed properly using the mounting medium. Images were taken under a light microscope. The relative expression of Nrf2 and caspase-3 proteins were measured using ImageJ software 1.48 version (NIH) (Java 1.8.9___66).

#### 4.6.12. Determination of Sciatic Nerve Structural Damage

The sciatic nerves were purged and placed at −80 °C on day 14 after PSNL for the spectroscopic analysis to check the lipid content of the myelin sheath in the PSNL model. The absorption spectrum is a plot as a function of wavenumber (ṽ) and is defined as the number of waves in a length of one centimeter and expressed in terms of cm^−1^. It is a widely used spectroscopic unit [108].The absorption spectrum is very complex since different vibrations react to the infrared light simultaneously. The vibration modes of each group are very sensitive alterations in the environment, chemical structure, and conformation of the molecule. Besides, the infrared spectrum of a certain molecule is unique since it consists of a unique combination of atoms. Therefore, the analysis of an infrared spectrum provides effective results in terms of the identification of materials. Moreover, it is commonly used in different branches of science since infrared spectroscopy is a non-destructive, quantitative qualitative technique [60]. The sciatic nerve samples were lyophilized and were used directly in the Fourier transform infrared (FTIR) spectrometer [109]. Moreover, the instrument was constantly removed from dry air to dismiss water vapors. The infrared spectra of sciatic nerve specimens were acquired in the FTIR spectrometer IR tracer (Shimadzu, Japan). The specimens were subjected to the range of 650 to 4000 cm^−1^ with a resolving power of 4 cm^−1^. Essential FTIR was used for digital data manipulation. For the estimation of the position of bands, the wavenumber values coinciding with the mid of weight were utilized.

#### 4.6.13. Statistical Analysis

Sigma plot version 12.5 statistical software (SYSTAT SOFTWARE, INC. USA) was used for the statistical analysis. Data from behavior and neurochemical analyses were expressed as mean ± standard deviations (S.D.). Normality and equality of variance were confirmed using Shapiro–Wilk’s and Brown-Forsythe’s tests respectively. Data from behavioral and biochemical experiments were analysed by using two-way analysis of variance (two-way ANOVA) followed by following Dunnett’s *t*-tests. Likewise, data from immunohistochemistry was analysed by using a one-way analysis of variance (one-way ANOVA) followed by Bonferroni’s Post hoc test. A Nonparametric Mann-Whitney U test (Minitab software) was used to analyse the differences in means of spectral data. For the statistical significance, the value of “P” less than 0.05 was selected as a criterion of significance difference.

## 5. Conclusions

**Figure 13:**
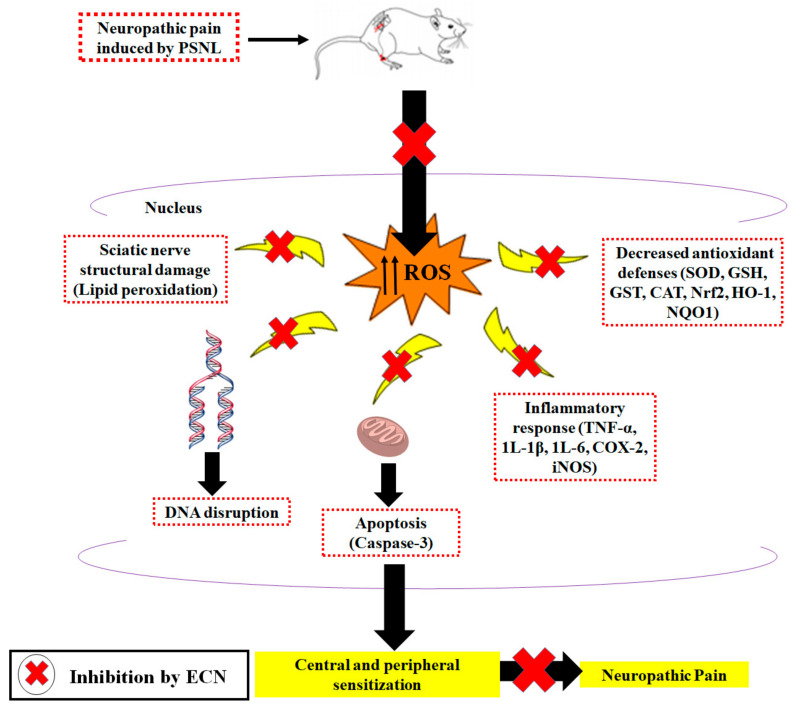
Proposed mechanism of the anti-neuropathic effect of ECN in the PSNL model.

In conclusion, the results of the present study indicated that ECN treatment significantly alleviates distress symptoms, mechanical allodynia, cold allodynia, mechanical hyperalgesia, and thermal hyperalgesia in the PSNL-induced model. ECN alleviated neuropathic pain in mice via inhibition of oxidative stress, inflammatory mediators, and pro-apoptotic proteins (Figure 13). Furthermore, it showed a protective effect on the lipid content of myelin sheath and prevented PSNL-induced histopathological alteration in the brain, spinal cord, and sciatic nerve. ECN seems to be reasonably safe as results of liver/renal function test and muscle activity. Unlike opioids and nonsteroidal anti-inflammatory drugs, minimal side effects and improved safety profile of ECN might contribute towards attenuating the pathological condition of neuropathic pain. The present study demonstrated that ECN could be a potential candidate for further development as a treatment option for chronic pain. Yet, additional research is worth to be investigated.

## Figures and Tables

**Table 1:** Effect of PSNL on liver and kidney functions.

Parameters	ALT (IU/L)	AST (IU/L)	Creatinine (mg/dL)
**Naive**	41.1 ± 2.9	45.5 ± 3.3	1.2 ± 0.25
**PSNL Control**	46.4 ± 5.3	48.9 ± 4.5	1.6 ± 0.31
**ECN (10 mg/kg)**	44.6 ± 4.2	47.1 ± 5	1.41 ± 0.27

Values are expressed as the mean S.D (n = 15 mice/group).

**Table 2:** The changes in the band wavenumber values of sciatic nerve spectra in the range of 3025–2800 cm^−1^

Bands	Naive	Sham Control	PSNL Control	PSNL + Pregabalin	PSNL + ECN	P
**Olefinic**	3008.9 ± 0.04	3004.2 ± 1.96	2999.3 ± 0.57	3003.2 ± 0.39	3002.2 ± 0.39	***
**CH_2_ antisymmetric**	2926.9 ± 1.59	2924.5 ± 2.11	2918.0 ± 0.90	2921.2 ± 0.35	2920.3 ± 0.34	***
**CH_2_ symmetric**	2857.23 ± 1.14	2854.6 ± 2.46	2848.0 ± 0.88	2853.3 ± 0.4	2852.1 ± 0.26	***
**CH_3_ antisymmetric**	2958.7 ± 0.13	2955.6 ± 3.14	2851.2 ± 1.07	2950.4 ± 0.51	2948.1 ± 1.01	***

A Nonparametric Mann-Whitney U test (Minitab software) was used to analyse the differences in means of the spectral data. Different letters meant statistically significant differences: (***) *p* < 0.001 indicate significant differences from the PSNL control group.

**Table 3:** General Band Assignment table for Sciatic nerve [63,64].

Wavenumber (cm^−1^)	Definition of the Spectral Assignment
**3014**	Olefinic=CH stretching vibration: unsaturated lipids, cholesterol esters.
**2962**	CH_3_ antisymmetric stretching: equal contribution of lipids, and proteins, carbohydrates, nucleic acids.
**2929**	CH_2_ antisymmetric stretching: mainly lipids, with the little contribution from proteins, carbohydrates, nucleic acids
**2855**	CH_2_ symmetric stretching: mainly lipids, with the little contribution from proteins, carbohydrates, nucleic acids

## Supplementary Materials

The following are available online, Figure S1: Effect of ECN (10 mg/kg) on MDA level on 7, 10, and 14-day post PSNL surgery in the hippocampus, prefrontal cortex, sciatic nerve and lumbar spinal cord of mice. MDA level expressed as percentage. Figure S2: Effect of ECN (10 mg/kg) on NO production on 7, 10- and 14-day post PSNL surgery in hippocampus, prefrontal cortex, sciatic nerve and lumbar spinal cord of mice. NO level expressed as percentage, Figure S3: Effect of ECN (10 mg/kg) on reduced SOD level on 7, 10- and 14-day post PSNL surgery in hippocampus, prefrontal cortex, sciatic nerve and lumbar spinal cord of mice. SOD level expressed as percentage, Figure S4: Effect of ECN (10 mg/kg) on GSH level on 7, 10- and 14-day post PSNL surgery in hippocampus, prefrontal cortex, sciat-ic nerve and lumbar spinal cord of mice. GSH level expressed as percentage, Figure S5: Effect of ECN (10 mg/kg) on GST level on 7, 10- and 14-day post PSNL surgery in hippocampus, prefrontal cortex, sciatic nerve and lumbar spinal cord of mice. GST level expressed as percentage, Figure S6: Effect of ECN (10 mg/kg) on catalase level on 7, 10, and 14 day post PSNL surgery in hippocampus, prefrontal cortex, sciatic nerve and lumbar spinal cord of mice, Figure S7: Effect of PSNL on histopathological changes in the kidney and liver of mice (H&E, × 10) (scale bar 50 μm). (A) In histo-pathological studies of kidney, photomicrograph of the PSNL control showing no histopathological alteration. (B) In his-topathological studies of liver, photomicrograph of the PSNL control showing normal hepatocytes with no histopatho-logical alteration.

## Sample Availability

Not available.
