# Supplementary material for: 7β-(3-Ethyl-cis-crotonoyloxy)-1α-(2-methylbutyryloxy)-3,14-dehydro-Z Notonipetranone Attenuates Neuropathic Pain by Suppressing Oxidative Stress, Inflammatory and Pro-Apoptotic Protein Expressions
Source: Molecules. 2021 Jan 1;26(1):181. doi: 10.3390/molecules26010181 (PMC7795484; doi:10.3390/molecules26010181)
Supplement: Supplementary file 1 [file molecules-26-00181-s001.pdf]

Supplementary Material

7β-(3-Ethyl-*cis*-crotonoyloxy)-1α-(2-methylbutyryloxy)-3,14-dehydro-*Z* Notonipetranone Attenuates Neuropathic Pain by Suppressing Oxidative Stress, Inflammatory and Pro-Apoptotic Protein Expression

Amna Khan [1], Adnan Khan [1], Sidra Khalid [1], Bushra Shal [1], Eunwoo Kang [2], Hwaryeong Lee [2], Geoffroy Laumet [3], Eun Kyoung Seo [2,*] and Salman Khan [1,*]

[1] Department of Pharmacy, Faculty of Biological Sciences, Quaid-i-Azam University, Islamabad, Pakistan; amna.khn26@gmail.com (A.K.); adkhan165sbbu@gmail.com (A.K.); sidra.merlin@gmail.com (S.K.); bushra.shal@gmail.com (B.S.)

[2] College of Pharmacy, Graduate School of Pharmaceutical Sciences, Ewha Womans University, Seoul 03760, Korea; smileunu@gmail.com (E.K.); jongsky119@naver.com (H.L.)

[3] Department of Physiology, Michigan State University, East Lansing, MI, USA; laumetge@msu.edu

* Correspondence: yuny@ewha.ac.kr (E.K.S.); skhan@qau.edu.pk (S.K.); Tel.: +82-2-3277-3047 (E.K.S.); +92-51-90644056 (S.K.)

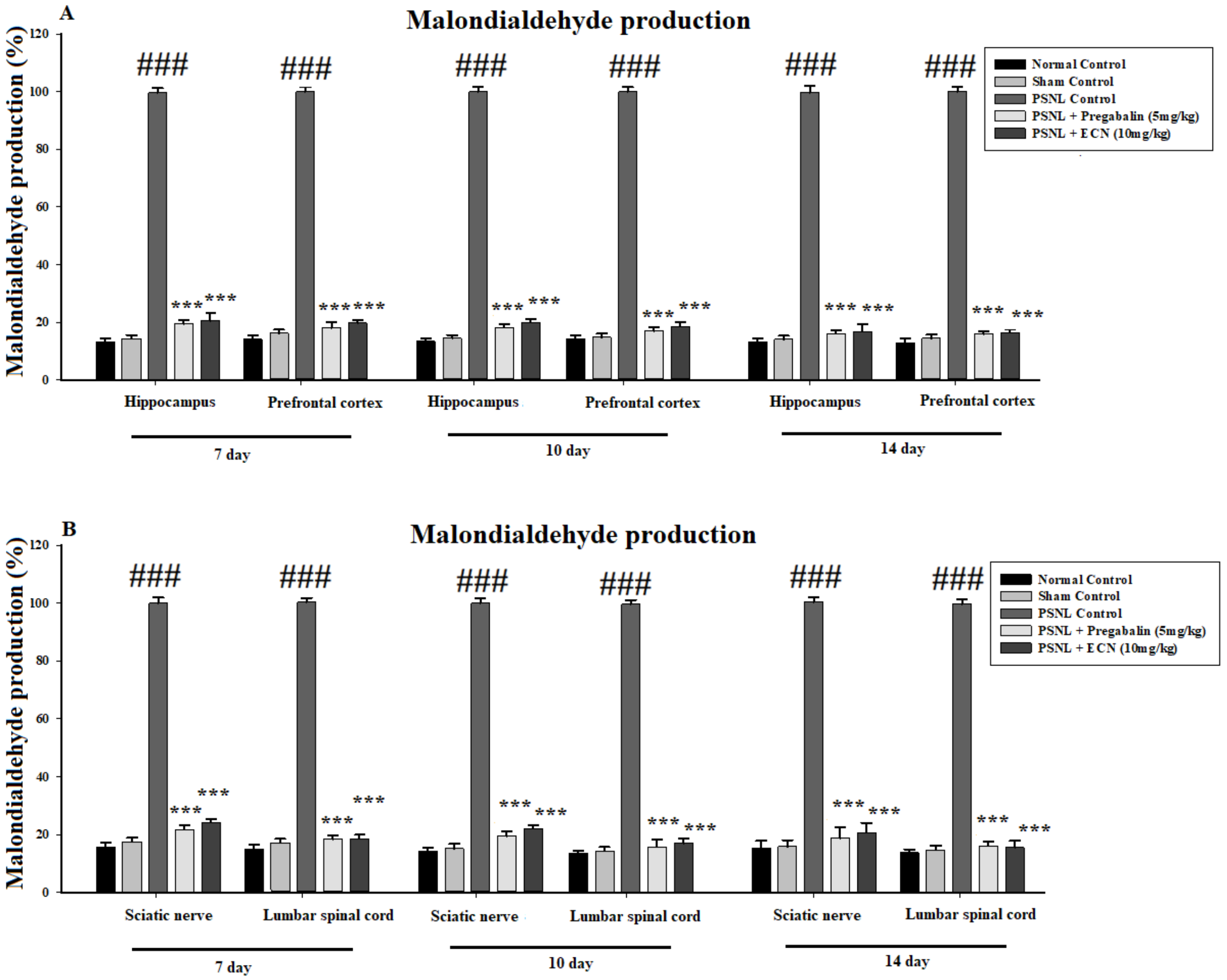

Figure S1. Effect of ECN (10 mg/kg) on MDA level on 7, 10, and 14 day post PSNL surgery in the hippocampus, prefrontal cortex, sciatic nerve and lumbar spinal cord of mice. MDA level expressed as percentage. The data is presented as the mean (n=3) ± S.D. Different letters meant statistically significant differences: (*) $P < 0.05$, (**) $P < 0.01$ and (***) $P < 0.001$ indicate significant differences from the PSNL control group. (###) indicate significant differences from the normal control group. ***$P < 0.001$ (two-way ANOVA followed by Dunnett's test).

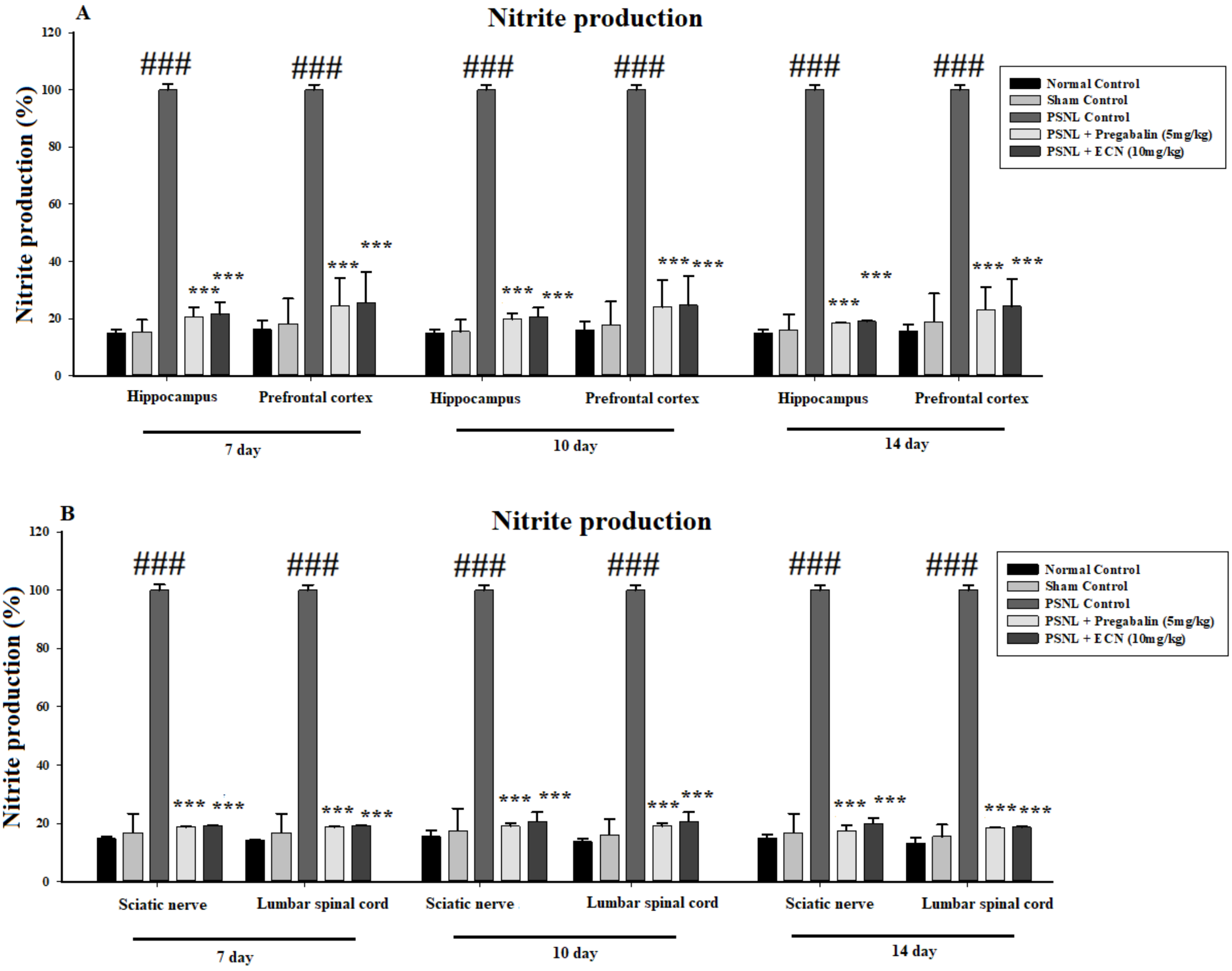

Figure S2. Effect of ECN (10 mg/kg) on NO production on 7, 10 and 14 day post PSNL surgery in hippocampus, prefrontal cortex, sciatic nerve and lumbar spinal cord of mice. NO level expressed as percentage. The data is presented as the mean (n=3) ± S.D. Different letters meant statistically significant differences: (*) P < 0.05, (**) P < 0.01 and (***) P < 0.001 indicate significant differences from the PSNL control group. (###) indicate significant differences from the normal control group. ***P < 0.001 (two-way ANOVA followed by Dunnett's test).

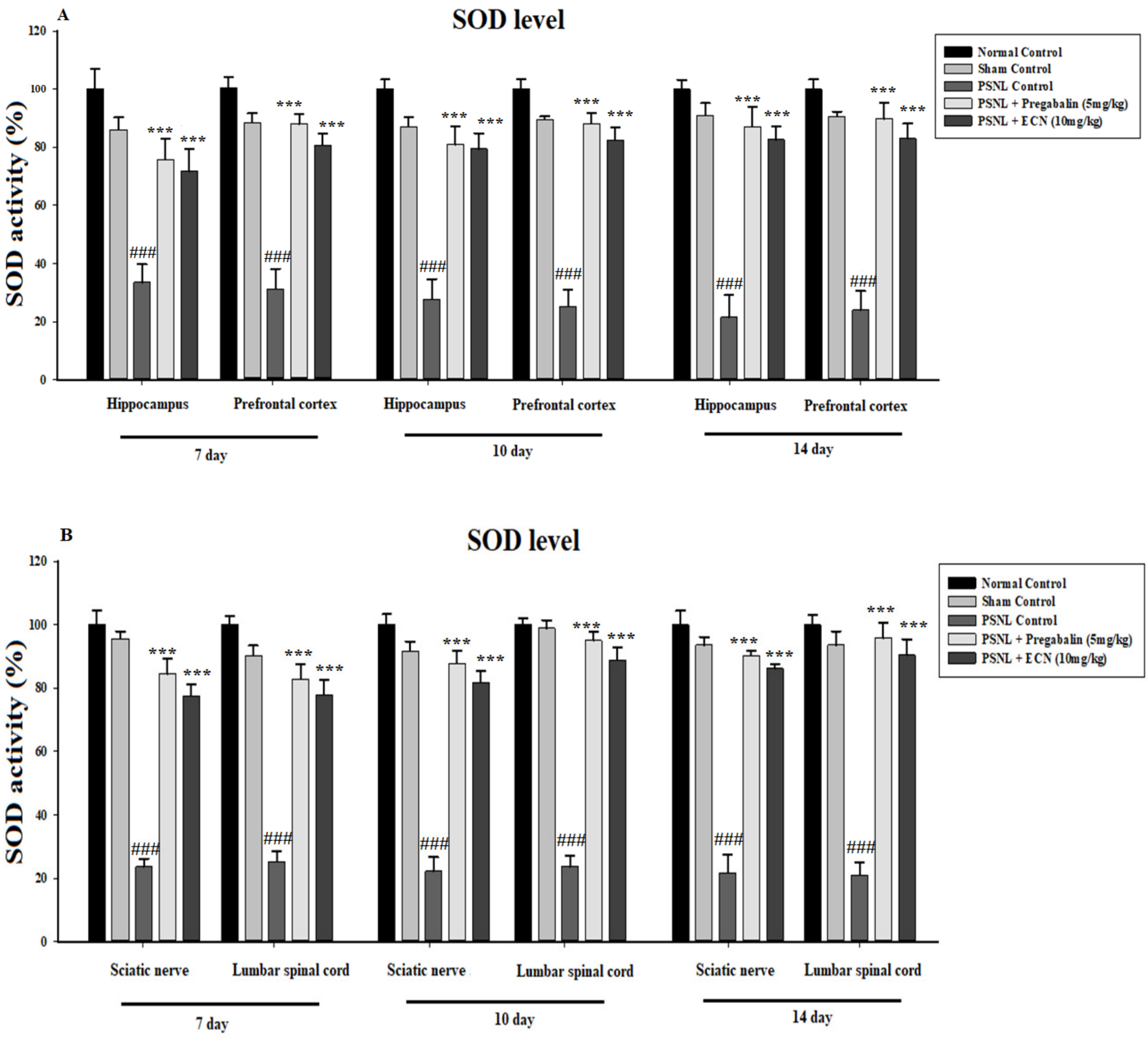

Figure S3. Effect of ECN (10 mg/kg) on reduced SOD level on 7, 10 and 14 day post PSNL surgery in hippocampus, prefrontal cortex, sciatic nerve and lumbar spinal cord of mice. SOD level expressed as percentage. The data is presented as the mean (n=3) ± S.D. Different letters meant statistically significant differences: (*) $P < 0.05$, (**) $P < 0.01$ and (***) $P < 0.001$ indicate significant differences from the PSNL control group. (###) indicate significant differences from the normal control group. ***$P < 0.001$ (two-way ANOVA followed by Dunnett's test).

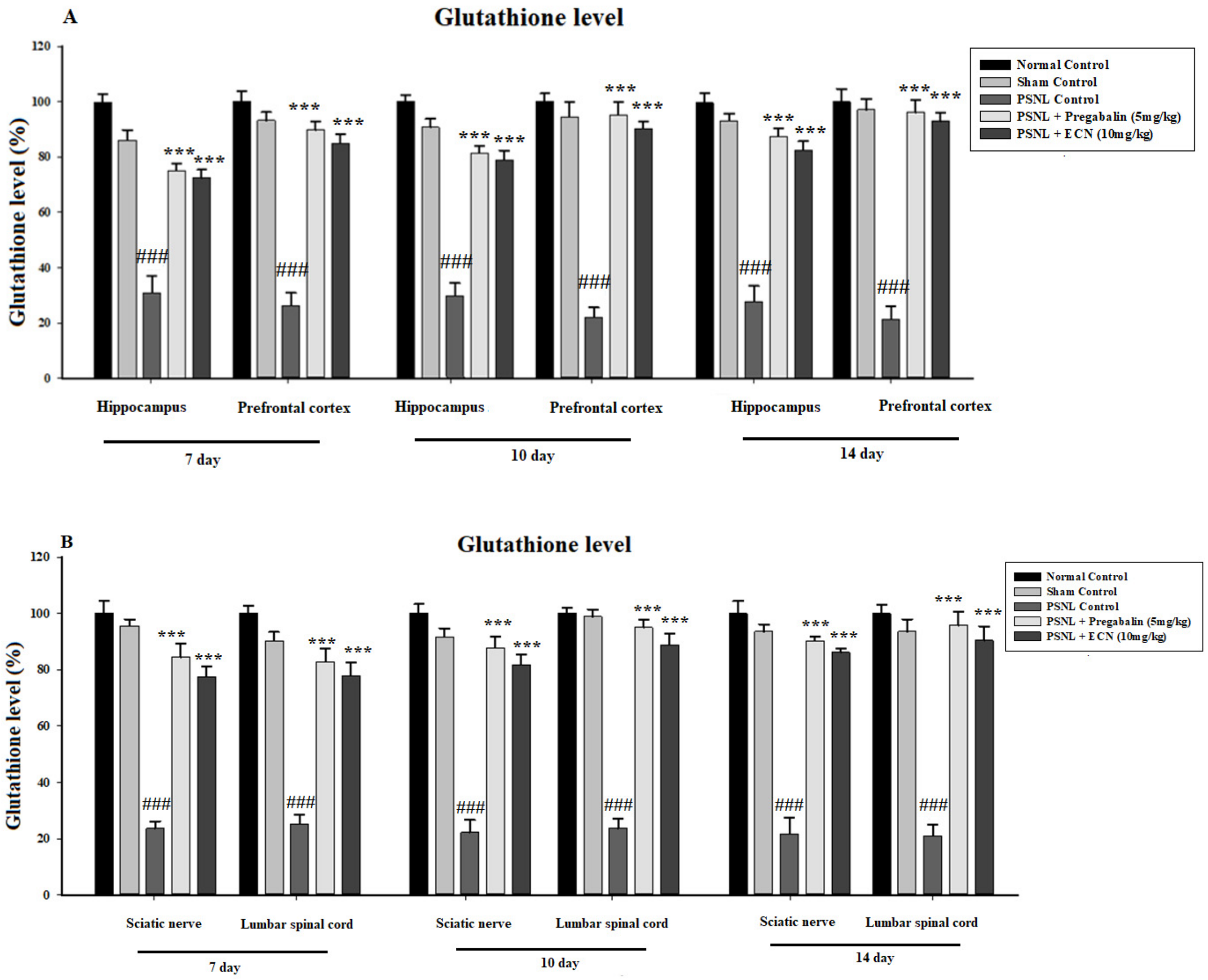

Figure S4. Effect of ECN (10 mg/kg) on GSH level on 7, 10 and 14 day post PSNL surgery in hippocampus, prefrontal cortex, sciatic nerve and lumbar spinal cord of mice. GSH level expressed as percentage. The data is presented as the mean (n=3) ± S.D. Different letters meant statistically significant differences: (*) $P < 0.05$, (**) $P < 0.01$ and (***) $P < 0.001$ indicate significant differences from the PSNL control group. (###) indicate significant differences from the normal control group. ***$P < 0.001$ (two-way ANOVA followed by Dunnett's test).

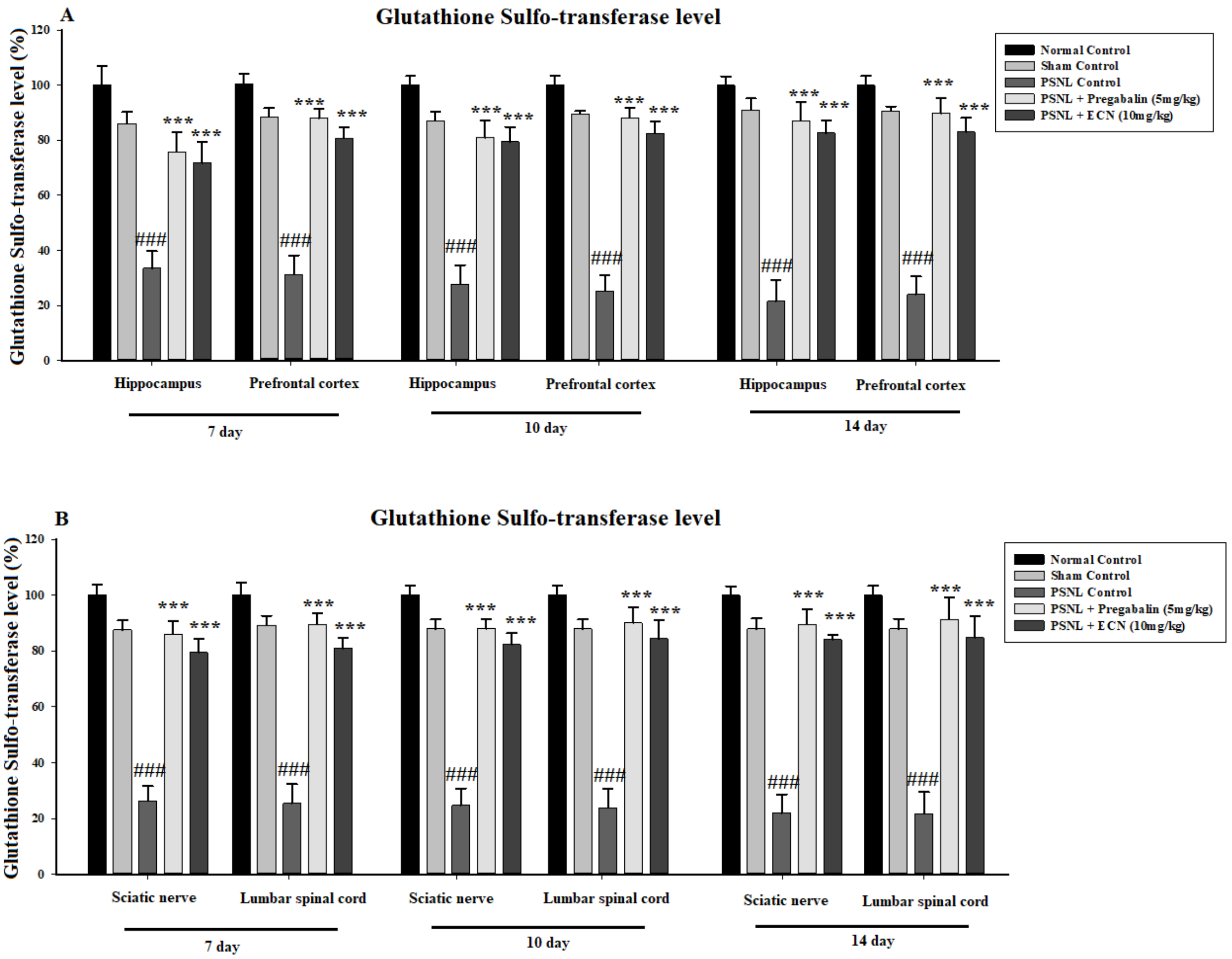

Figure S5. Effect of ECN (10 mg/kg) on GST level on 7, 10 and 14 day post PSNL surgery in hippocampus, prefrontal cortex, sciatic nerve and lumbar spinal cord of mice. GST level expressed as percentage. The data is presented as the mean (n=3) ± S.D. Different letters meant statistically significant differences: (*) $P < 0.05$, (**) $P < 0.01$ and (***) $P < 0.001$ indicate significant differences from the PSNL control group. (###) indicate significant differences from the normal control group. ***$P < 0.001$ (two-way ANOVA followed by Dunnett's test).

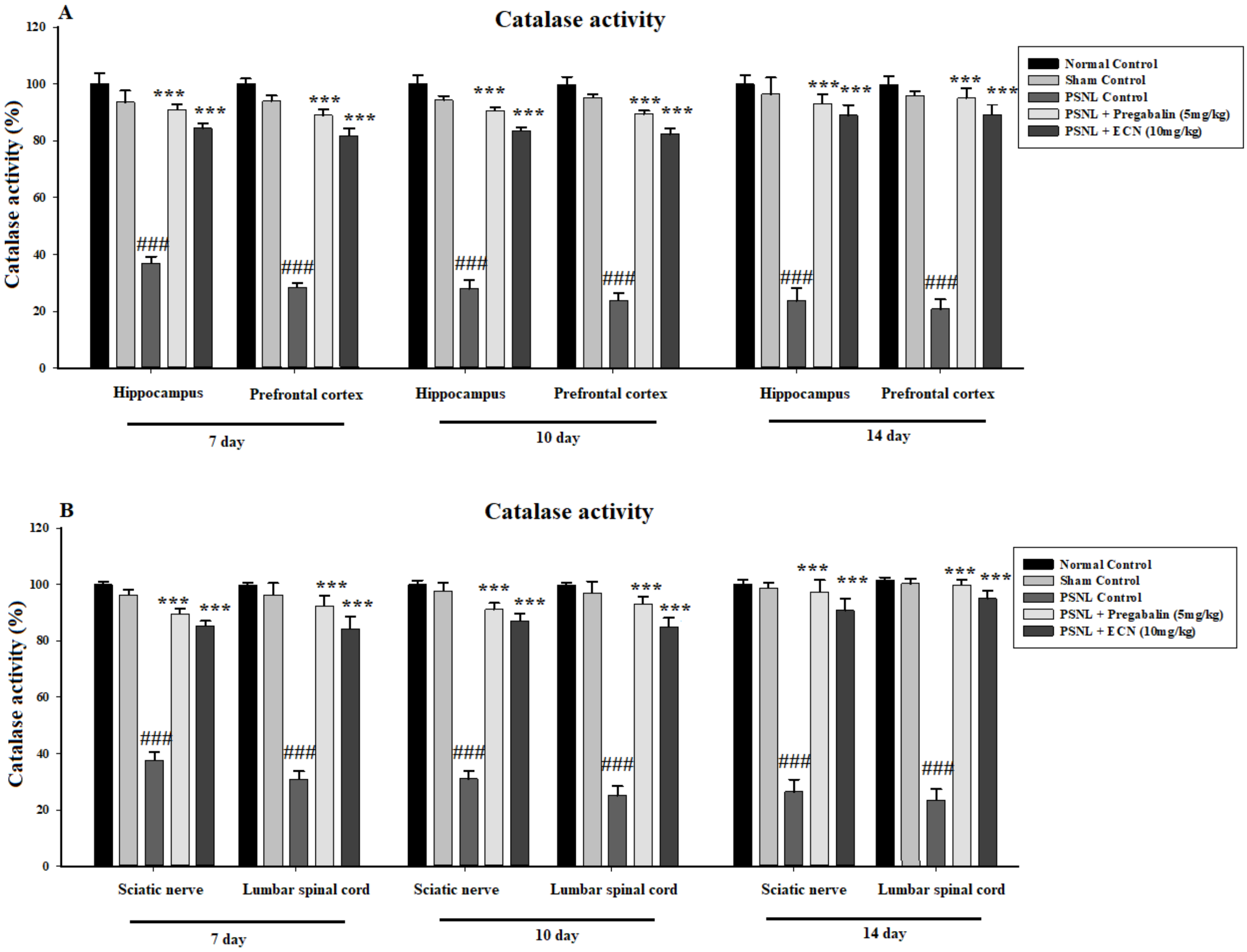

Figure S6. Effect of ECN (10 mg/kg) on catalase level on 7, 10, and 14 day post PSNL surgery in hippocampus, prefrontal cortex, sciatic nerve and lumbar spinal cord of mice. Catalase level expressed as percentage. The data is presented as the mean (n=3) ± S.D. Different letters meant statistically significant differences: (*) P < 0.05, (**) P < 0.01 and (***) P < 0.001 indicate significant differences from the PSNL control group. (###) indicate significant differences from the normal control group. ***P < 0.001 (two-way ANOVA followed by Dunnett's test).

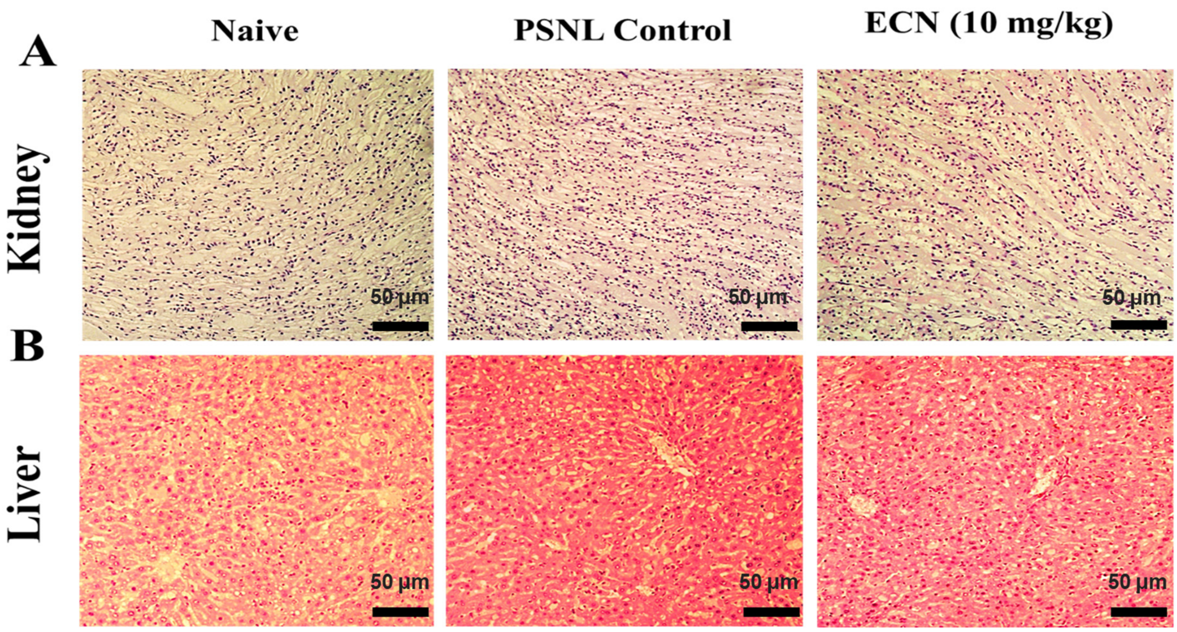

Figure S7. Effect of PSNL on histopathological changes in the kidney and liver of mice (H&E, ×10) (scale bar 50 µm). (**A**) In histopathological studies of kidney, photomicrograph of the PSNL control showing no histopathological alteration. (**B**) In histopathological studies of liver, photomicrograph of the PSNL control showing normal hepatocytes with no histopathological alteration.
